# Phenotypic variation of *Chitala chitala* (Hamilton, 1822) from Indian rivers using truss network and geometric morphometrics

**DOI:** 10.7717/peerj.13290

**Published:** 2022-04-18

**Authors:** Rejani Chandran, Achal Singh, Rajeev K. Singh, Sangeeta Mandal, Kantharajan Ganesan, Priyanka Sah, Pradipta Paul, Abhinav Pathak, Nimisha Dutta, Ramashankar Sah, Kuldeep K. Lal, Vindhya Mohindra

**Affiliations:** 1 Fish Conservation Division, National Bureau of Fish Genetic Resources, Lucknow, Uttar Pradesh, India; 2 Department of Fisheries, Bankura, West Bengal, India; 3 Molecular Biological Sciences, Farelabs Private Limited, Gurugram, India

**Keywords:** Conservation, Intra-specific, Management, Plasticity, Population structure, Variation

## Abstract

*Chitala chitala* (Hamilton, 1822) is an economically important food fish species occurring throughout Indian rivers, which also has ornamental value. This study focuses on morphological variations in *C. chitala* from seven river basins across India namely; Son, Tons, Ken, Brahmaputra, Ganga, Gomti and Gandak. A truss network was constructed by interconnecting nine landmarks to generate 36 morphometric variables extracted from digital images of specimens sampled from the study locations. Transformed truss measurements were subjected to principal component analysis (PCA), canonical discriminant function analysis (CDFA) and discriminant analyses of principal components (DAPC). DAPC function coefficients performed much better in capturing the variation pattern and discrimination between the rivers which was not achieved using CDFA. Eight truss variables were identified with significant and highest loading for truss variables on principal components and coefficients on discriminant function from DAPC contributing to maximum variation between the rivers. Performance graph and functional distribution of identified truss variables clearly indicated distinction between the rivers. Thin plate spline analysis and procrustes shape analysis further showed the variation in morphology between specimens across the rivers. The significant parameters differentiating specimens from different rivers were linked to dorsal fin origin, the base of the pectoral fin and the perpendicular point on the anal fin from the dorsal fin origin. Variation in the hydrodynamics of the rivers studied might be possibly affecting the fin kinematics and consequently leading to adaption seen as phenotypic variation in *C. chitala*. The results showcased in the present study shall help in better understanding of intra-specific diversity which is significant for management and conservation of a species.

## Introduction

The Clown Knifefish, *Chitala chitala* (Hamilton, 1822) of family Notopteridae, belongs to one of the primitive orders, Osteoglossiformes. The fossil records of order Osteoglossiformes have been retrieved from the deposits belonging to late Jurassic or early Cretaceous period indicating an ancestral teleost lineage (Hilton & Lavoue, 2018). The long anal fin which is confluent with caudal fin gives an appearance of feather; due to which *C. chitala* is commonly known as humped featherback (Chonder, 1999). One of the studies on genetic diversity using molecular and protein markers, indicated the possibility of ancestors of the *C. chitala* surviving the pre-historical desiccation period and passing through genetic bottleneck (Mandal et al., 2009). *C. chitala* inhabits riverine waters but can adapt in stagnant water due to their swim bladder modification, which functions as an accessory respiratory organ (Mitra, Mukhopadhyay & Homechaudhuri, 2018). In India, it inhabits the Mahanadi and Ganga–Brahmaputra river basins as well as swamps and is largely popular for its flesh quality and ornamental value (Chandran et al., 2020; Dutta et al., 2020). *C. chitala* is a high-priced fish for food, sport and aquarium purposes due to its rarity and delicacy (Gopalan, Ramasastri & Balasubramanian, 2004; Sarkar, Deepak & Lakra, 2009). The population of this species has reduced considerably due to over-exploitation, habitat alteration, pollution and related anthropogenic pressure on their natural habitats and thus, is categorized as near threatened (NT) by International Union for Conservation of Nature (Swain et al., 2021) though it was earlier categorized as endangered (EN) (Ayyappan, Raizada & Reddy, 2001; Sarkar et al., 2007). Assessment of *C. chitala* for the IUCN Red List of Threatened Species was most recently carried out by Chaudhry (2010).

The diversity information below species level, though lacking in wild relatives of most aquaculture species, has been considered as an important knowledge milestone for enhancing the utilization of farmed types for food production in the future (FAO, 2019). In order to manage wild populations, effectively contribute to fisheries and for aquaculture improvement, it is important to understand intra-specific diversity of the species (Rawat et al., 2017). Mostly the aquaculture species and their wild relatives have been characterized using various molecular tools (Benzie et al., 2012). Several genetic studies have been carried out in *C. chitala* based on allozyme (Mandal et al., 2009), mitochondrial (Mandal et al., 2012; Dutta et al., 2020; Anjarsari et al., 2021) and microsatellite markers (Dutta et al., 2020). Dutta et al. (2020) has identified four genetic stocks, *viz.,* Ganges–Brahmaputra, Sutlej, Mahanadi and Narmada, in *C. chitala* from 14 rivers across India; by combined analyses of two full mitochondrial genes, *ATPase 6/8* and *Cytochrome b*.

Management units justify management of population within species separately due to genetic differentiation (Robalo et al., 2021). Morphological variation between populations together with molecular data (Camelier et al., 2018; Ukenye, Taiwo & Anyanwu, 2019) can play an important role in understanding variation. Phenotyping research on fish species to define intra-specific diversity is still at infancy and has not been widely applied for most of the aquaculture and harvested species. An integrated approach, including both phenotypic and genetic stock are necessary for thorough population characterisation (Swain & Foote, 1999). The present study aimed to find out the existence of phenotypic stock, if any, by examining the body shape differences to identify phenotypic variations and divergence, using truss network system and geometric morphometrics in the population of *C. chitala* in seven rivers from Ganga and Brahmaputra basins of India as Dutta et al. (2020) had identified a single genetic stock from these rivers. Truss network profiles generated by the use of landmarks extending across the shape of entire fish captures shape information and provide a quantitative method to assess morphometric differences between the specimens from different geographical locations (Strauss & Bookstein, 1982; Turan, 1999; Ethin et al., 2019; Mahfuj, Rahman & Samad, 2019). There is need for scientific studies on a variety of species to understand the utility of truss network analysis, to be used as a marker or tool in determining the intra-specific phenotypic diversity. The present study also compares the power of different statistical techniques, based on truss and cartesian landmark data in resolution of spatial morphological diversity of the species. The paper also explains the functional morphology of specific landmarks and morphometric characters which are observed to have significant contribution to diversity with relevance to biological adaptations in *C. chitala*.

## Material and Methods

### Collection of samples

A total of 149 dead specimens of *C. chitala* (a near threatened species) were collected from commercial riverine catches of seven rivers across India, *viz,* Son (*n* = 43), Tons (*n* = 16), Ken (*n* = 31), Brahmaputra (*n* = 16), Ganga (*n* = 13), Gomti (*n* = 20) and Gandak (*n* = 10), at a stretch from May, 2009 to November 2017 with repeated collections from a particular location to achieve a reasonable sample size (Table 1, Fig. S1). These samples form a subset of those studied by Dutta et al. (2020) through mitochondrial markers and had identified a single genetic stock from these rivers. No live animals were used in the experiment performed. All the fish, which were dead, were procured from commercial catches. The protocols followed were approved by the Institutional Animal Ethical Committee (IAEC), ICAR-NBFGR, Lucknow, India vide No. G/CPCSEA/IAEC/2015/2 dated 27 October 2015.

**Table 1:** Description of collection localities, number of samples (N) and time of collection of *Chitala chitala*.

Sl. No	River	Site	Coordinates (Longitude/Latitude)	Time of collection	N
1	Son	Bansagar, Beohari, Madhya Pradesh	80.88, 24.055	Jan. 2011 May 2012 May 2015 May 2016 April 2017	01 02 09 26 05
2	Tons	Rewa, Madhya Pradesh	81.17, 24.31	May 2009 May 2016 April 2017	04 07 05
3	Ken	Rangua, Madhya Pradesh	79.89, 24.69	Sep. 2015 June 2016 July 2017	14 08 09
4	Brahmaputra	Uzan Bazaar, Assam Dubri, Assam	91.75, 26.19 89. 58, 26.01	Nov. 2015 Nov. 2015	11 05
5	Ganga	Farakka, West Bengal	87.91063, 27.767695	April 2016 Nov. 2016	7 6
6	Gomti	Lucknow, Uttar Pradesh	80.95, 26.86	Oct. 2016 June 2017	14 06
7	Gandak	Valmiki Nagar, Bihar	84.327246, 26.78437	Nov. 2017	10
TOTAL					149

### Digitization of samples

**Figure 1:**
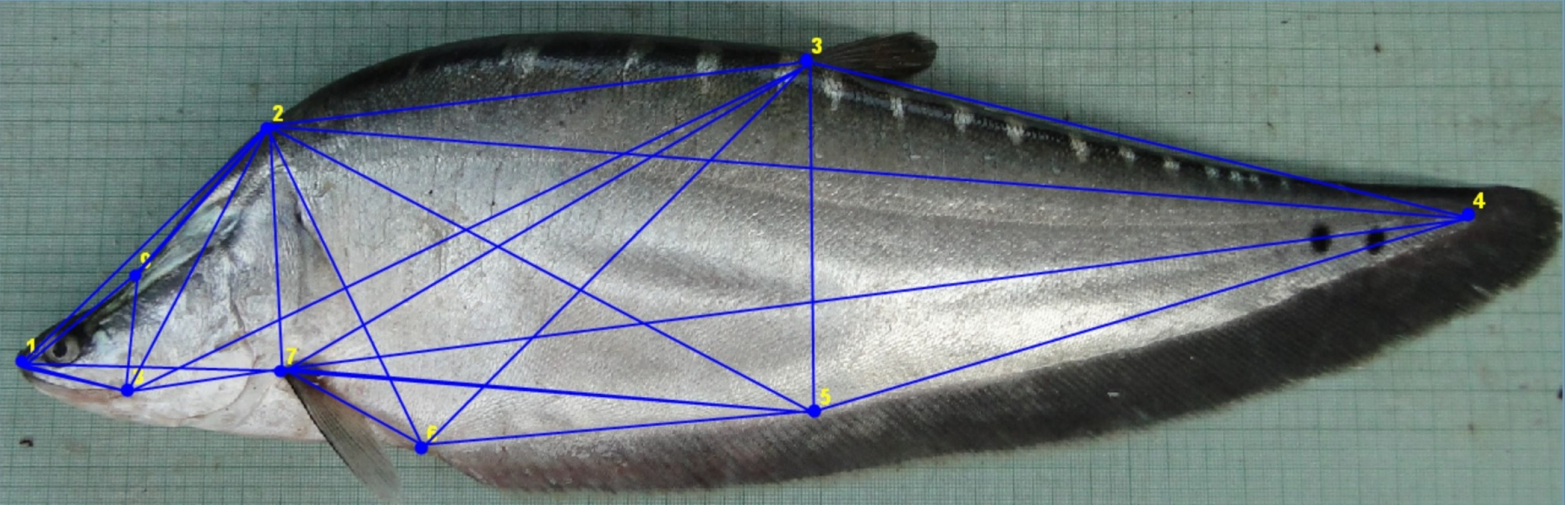
Truss network of *Chitala chitala* showing the nine landmarks used for morphometric analysis. 1. Anterior tip of snout at upper jaw; 2. posterior most aspect of neurocranium (beginning of scaled nape); 3. dorsal fin origin; 4. posterior end of vertebrae column; 5. line perpendicular to dorsal fin origin; 6. anal fin origin; 7. pectoral fin insertion; 8. posterior end of maxilla; 9. line perpendicular to posterior end of maxilla. Source credit: ICAR-NBFGR.

The specimens were first cleaned with water, drained, wiped dry and placed on a flat surface having laminated graph sheet on background, which was meant for calibration of the coordinates of digital images (Fig. 1). The methodology of capturing digital images for truss network analysis by Mandal et al. (2020) was followed. The fins were stretched for clear visibility of origin and insertion points. Each specimen, placed on graph paper, was labelled with a unique code and captured using DSC-W300 digital camera (Sony, Japan) with mouth facing towards left, which permits replication of the measurements (Cadrin & Friedland, 1999). Due to varying size range of samples as the images were captured from different heights, reference scale on the graph paper was used for calibration, using the software tpsUtil (Rohlf, 2008a).

### Generation of morphometric data

Nine landmarks were identified that extended across the entire fish to represent the full dimensions of the morphology (Fig. 1) and truss data was generated from digitized images through tpsUtil V1.38 (Rohlf, 2008a) program for converting the images secured from JPEG (*.jpeg) to TPS (*.tps). The truss distances were extracted in tps files from the digitised images using tpsDig2 ver. 2.31 in X-Y coordinate data form (Rohlf, 2008b). PAleontological STatistics (PAST) software (Hammer, Harper & Ryan, 2001) was used to construct an inter-linked network of nine landmarks to generate 35 truss measurements.

### Statistical analysis

The landmark-geometric morphometric (GM) analysis were performed on truss –morphometric data after eliminating size-effect. The data generated by PAST were log-transformed (Strauss, 1985) for linearization of data and to minimize size-related dissimilarity, all morphometric variables were adjusted by an allometric approach as proposed by Elliott, Haskard & Koslow (1995); M_adj_ =M*(L_S_/L_0_)^b^, where M = original measurement, M_adj_ = size adjusted measurement, L_0_ = standard length of fish, L_S_ = overall mean of standard length for all fish from all locations. As standard length (SL, character code 1_4) was used as a basis for transformation, it was excluded from the final analysis (Mamuris et al., 1998) and thus 35 morphometric variables were retained. One-way ANOVA for 35 morphometric characters was performed to evaluate the significant difference among seven rivers. The morphometric characters which showed high significant variations (*P* < 0.01) were used to obtain the recommended ratio of the number of samples (N) to parameters included (P), *i.e.,* 3.5 to 8, for a stable outcome from multivariate analysis (Johnson, 1981; Kocovsky, Adams & Bronte, 2009). Therefore, 149 fish specimens of *C. chitala,* which is a subset of specimens collected for genetic stock structure analysis (Dutta et al., 2020); with nine-landmarks and 36 truss variables were found to be optimum for GM analysis.

For assessing distribution pattern of variance associated with variables principal component analysis (PCA) was applied on data matrix of 149 × 35 by reducing redundancy among the morphometric variables and eventually eliminating abundant independent variables (Samaee, Patzner & Mansour, 2009). Kaiser’s (1960) criterion of retaining eigenvalues greater than one (Jolliffe, 2002) was applied to determine PCA components. The canonical discriminant function analysis (CDFA) and discriminant analysis of principal components (DAPC) was also applied on truss data, to identify important truss variables associated with discriminant functions and the distribution pattern of specimens over geographical locations through discriminant functions. CDFA was applied on truss data for identification of important discriminant functions, important truss variables associated with discriminant functions and the distribution pattern of specimens over locations through discriminant functions (Cadrin, 2000). PCA identifies variations within specimens while CDFA identifies variations in specimens over locations. Therefore, a technique (DAPC) was also employed to simultaneously identify the within and between location variations. Further, to assess sensitivity of discriminant function in discrimination of specimen over different locations, the shape variation through receiver operating characteristics (ROC) curve analysis over (1-specificity) *vs.* sensitivity as axis with values varying in range from (0, 0) to (1, 1) was also carried out. In this analysis, area under curve of ROC performance graph of truss variables, identified by DAPC, indicated the role of discriminant functions in differentiating among locations, rivers, environments. ArcMap 10.8.1 platform (2020) was used to represent the coefficient of variation (CV) of significant top four truss variables, identified by DAPC, on geo-spatial scale. The river network shape file was prepared using the Google Earth platform and river basin shape file was obtained from NRSC India-Water Resource Information System (https://indiawris.gov.in/wris/). To understand geometric shape variations and distribution of landmark configurations, geometric shape variations in mean shape of specimens were analysed through thin-plate-spline (TPS) image analysis (Rohlf, 2008c). This was employed through procrustes superimposition method to extract shape information and infer shape changes, based on the anatomical landmark coordinates. Along with this, generalised procrustes shape variations over principal components of landmark coordinates were also performed to understand overall body shape variation across all landmarks simultaneously. Set of nine landmarks identified were partitioned into two subsets to check for shape variation through procrustes shape modularity.

All statistical analyses for truss-morphometric data were performed using R (ver.4.1.2) packages Adegenet 2.15 & ggplot2, SPSS version 16 (SPSS Inc. SPSS for Windows), SAS version 9.3 (SAS Inc: 2020), PAST (ver. 3.0), MorphoJ (Klingenberg, 2011) and Excel (Microsoft Corporation, 2018).

## Results

### Principal component analysis (PCA)

**Figure 2:**
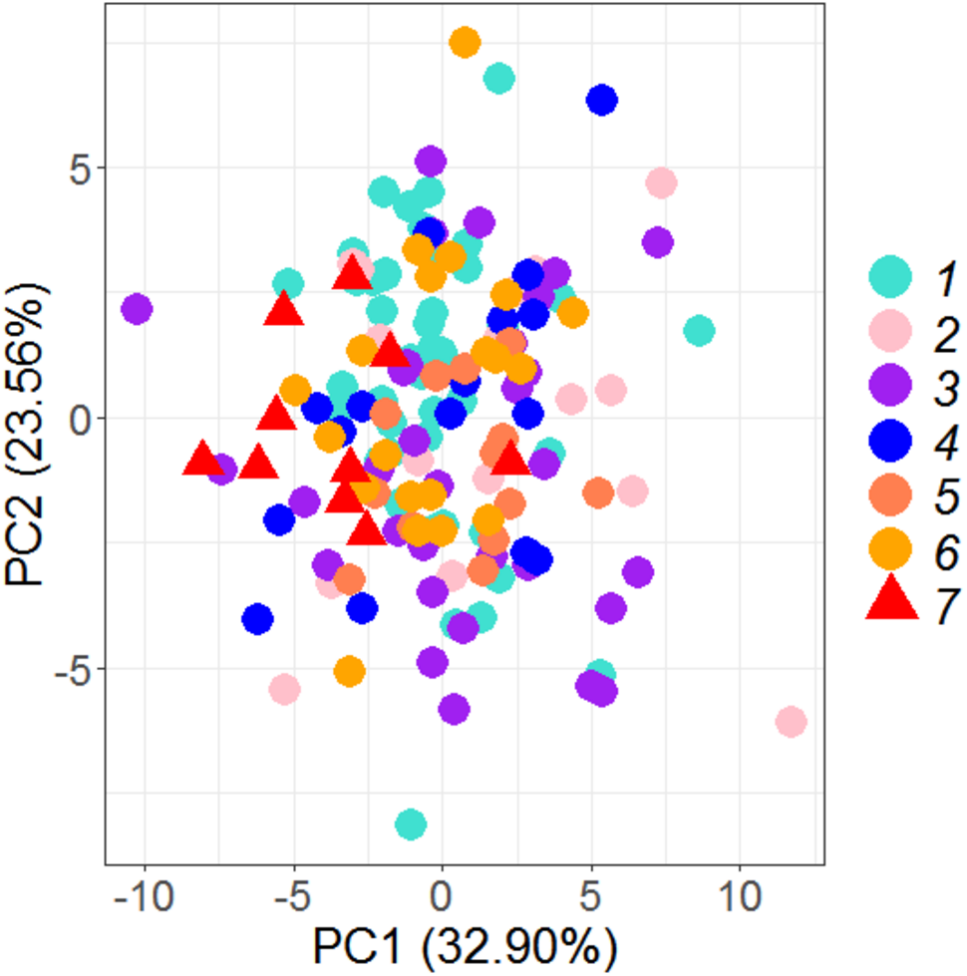
Specimen’s distribution over shape scores on principal components (PC1 *vs* PC2) in PCA analysis of data matrix 149 × 35. 1. Son; 2. Tons; 3. Ken; 4. Brahmaputra; 5. Ganga; 6. Gomti; 7. Gandak.

**Figure 3:**
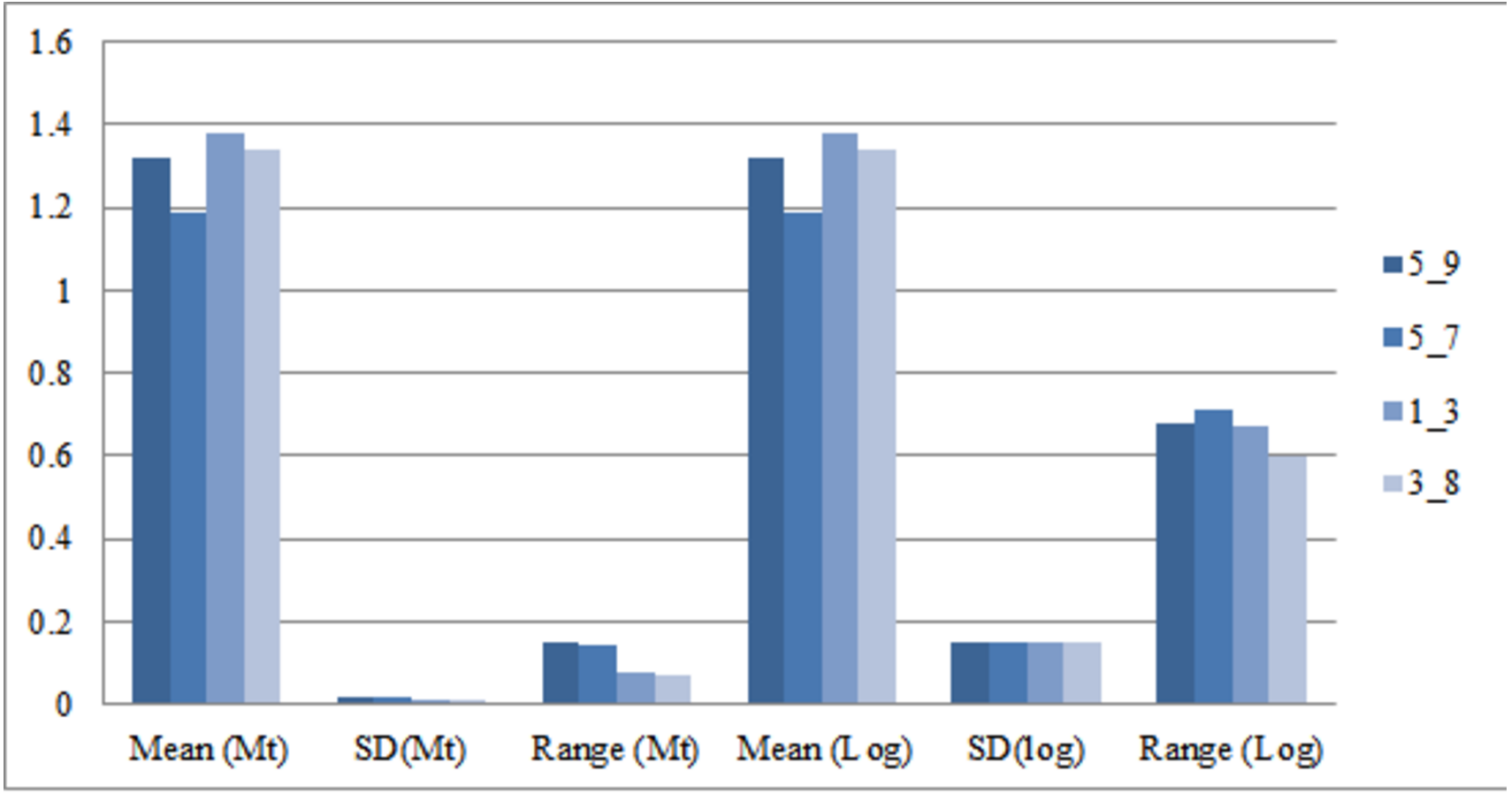
Truss-morpho linking over mean, standard deviation, range values of four identified truss variables in Mtrans data (Mt) and log-morphometric data (Log). 5_9: Distance between point perpendicular to dorsal fin origin and point perpendicular to posterior end of maxilla; 5_7: Distance between point perpendicular to dorsal fin origin and pectoral fin insertion; 1_3: Distance between anterior tip of snout at upper jaw and dorsal fin origin; 3_8: Distance between dorsal fin origin and posterior end of maxilla.

PCA on 35 morphometric characters on (*n* = 149) individuals from seven locations, resulted in identifying 6 significant principal components that contributed up to 93.23% of total variation (Table 2) and eight truss variables with significant and highest loadings on PC-1 and PC-2 (Figs. 2, 3, Table 3). The two PCs cumulatively accounted for 56.46% of total variation with PC-1 accounting for 32.90% that led to the identification of two most important truss variables 5_9 (distance between point perpendicular to dorsal fin origin and point perpendicular to posterior end of maxilla), and 5_7 (distance between point perpendicular to dorsal fin origin and pectoral fin insertion), with significant loadings (Tables 2 and 3).

### Relationship between truss and morphometric variables

Morphometric variation between significant truss measurements was identified after normalizing the data by transformation. As Elliot transformation accounts for only size related dissimilarity, an effort was made to compare variation using log transformed truss measurement too as it considers both shape and size dissimilarity. Comparison of the mean for truss variables in M-trans (Elliot transformation) ranged from 0.97–1.38 while in log-morphometric data it varied from 1.18–1.53 (Table 3). For instance, the truss variable, 5_9, in log-morphometric data exhibited higher standard deviation (0.15) and higher range value (0.68) than M-trans data (0.02, 0.15), with same value of mean (1.32). Thus, improved morphometric based identification using the truss variables in log-morphometric data is possible rather than M-trans due to higher standard deviation and range value indicating its greater utilization and application at field level (Table 3, Fig. 3).

### Canonical discriminant function analysis (CDFA)

**Figure 4:**
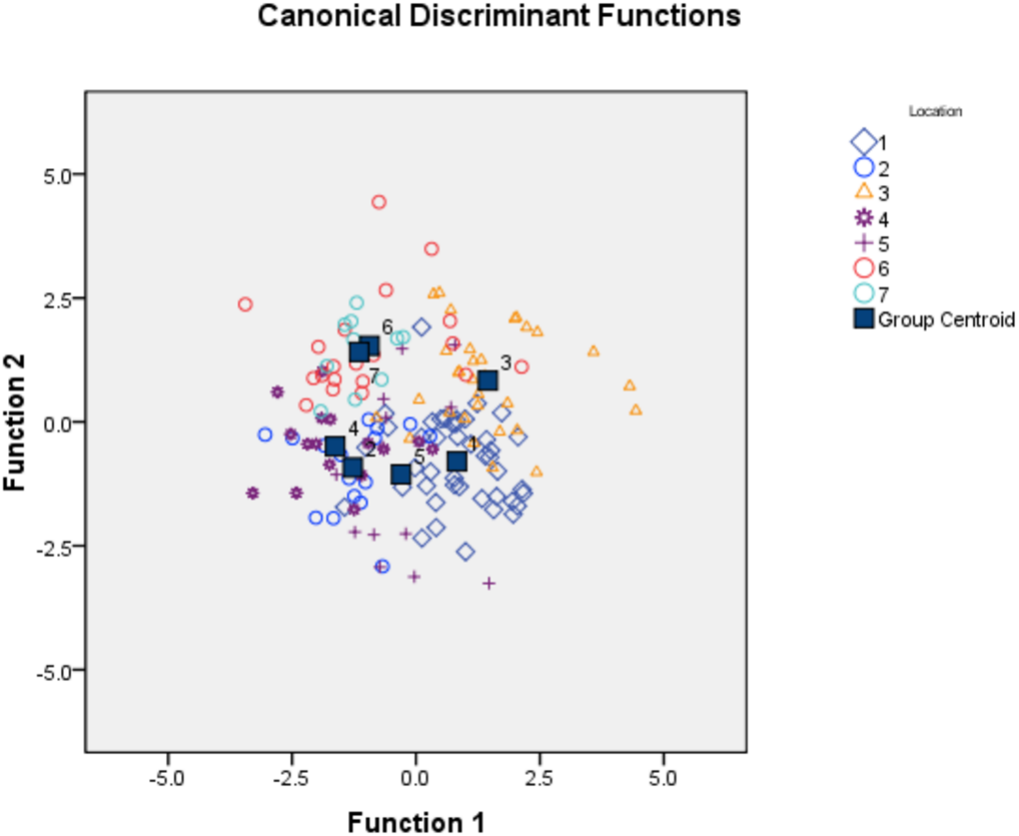
Specimens distribution from seven location as per canonical discriminant functions 1 and 2 in CDF analysis of data matrix 149 × 35. 1. Son; 2. Tons; 3. Ken; 4. Brahmaputra; 5. Ganga; 6. Gomti; 7. Gandak.

To identify important truss variables associated with discriminant functions, CDFA provided four canonical discriminant functions, which controlled 90.27% of variation with Wilks’ Lambda values ranging from 0.05–0.12, with significant chi-square value (*p* < 0.05). Two functions (1 & 2) out of the four, cumulatively explained 58.16% of total variation, where function-1 contributed the highest variation (33.06%) followed by function-2 (25.10%) (Fig. 4, Table 4). CDFA identified four most important truss variables with discrimination coefficient for 2_3 and 1_5 as 309.52 and 212.01 respectively for function-1 and for 3_9 and 4_8 as 582.49 and (-) 1034.82 for function-2, respectively. CDFA displayed significant differences among specimens from rivers Son, Ken and Ganga by distinct centroids, but similarity of specimen by merged centroids for river Tons with Brahmaputra and Gomti with Gandak (Fig. 4).

**Table 2:** Principal component analysis (PCA) in *Chitala chitala* for truss analysis.

Principal components	Eigen value	Percentage of variance	Cumulative variance
1	11.52	32.90	32.90
2	8.25	23.56	56.46
3	6.44	18.41	74.88
4	2.94	8.40	83.27
5	2.20	6.29	89.56
6	1.28	3.67	93.23

**Table 3:** Truss-morphometric relationship through mean, standard deviation (SD), minimum, maximum and range value of eight identified truss variables in *Chitala chitala*.

**Sl. No.**	**Truss variable**	**Truss variable loading on two principal components**		**Truss variables, M-trans data matrix (after eliminating the effect of standard length)**					**Truss variables Log-morphometric data matrix (without eliminating the effect of standard length)**				
		PC1*	PC2*	**Mean**	**SD**	**Min**	**Max**	**Range**	**Mean**	**SD**	**Min**	**Max**	**Range**
1	5_9	0.90	0.33	1.32	0.02	1.24	1.39	0.15	1.32	0.15	0.91	1.59	0.68
2	5_7	0.83	0.26	1.19	0.02	1.10	1.23	0.14	1.19	0.15	0.75	1.46	0.71
3	2_3	0.79	0.30	1.18	0.02	1.10	1.22	0.13	1.18	0.15	0.75	1.45	0.71
4	1_5	0.75	0.58	1.37	0.02	1.31	1.42	0.11	1.37	0.15	0.95	1.63	0.68
5	1_3	0.15	0.92	1.38	0.01	1.34	1.42	0.08	1.38	0.15	0.96	1.63	0.67
6	3_8	0.44	0.78	1.34	0.01	1.29	1.37	0.07	1.34	0.15	0.93	1.58	0.66
7	4_7	−0.73	0.66	0.97	0.04	0.87	1.09	0.23	1.53	0.14	1.13	1.79	0.66
8	4_5	−0.73	0.66	0.97	0.04	0.87	1.09	0.23	1.29	0.14	0.88	1.57	0.69

**Notes.**

* Loading of Components significant (>or <0.13 or −0.13).

SD, Standard deviation; Min, Minimum; Max, Maximum.

5_9: Distance between point perpendicular to dorsal fin origin and point perpendicular to posterior end of maxilla; 5_7: Distance between point perpendicular to dorsal fin origin and pectoral fin insertion; 2_3: Distance between posterior most aspect of neurocranium and dorsal fin origin; 1_5: Distance between anterior tip of snout at upper jaw and point perpendicular to dorsal fin origin; 1_3: Distance between anterior tip of snout at upper jaw and dorsal fin origin; 3_8: Distance between dorsal fin origin and posterior end of maxilla; 4_7: Distance between posterior end of vertebrae column and pectoral fin insertion; 4_5: Distance between posterior end of vertebrae column and point perpendicular to dorsal fin origin.

**Table 4:** Canonical discriminant function analysis (CDFA) in *Chitala chitala*.

Sl No	Centroid for river	Function-1	Function-2
1	Son	0.83	−0.80
2	Tons	−1.27	−0.92
3	Ken	1.45	0.83
4	Brahmaputra	−1.63	−0.49
5	Ganga	−0.30	−1.06
6	Gomti	−0.94	1.54
7	Gandak	−1.15	1.41
8	(%) variance	33.06	25.10
9	Cumulative variance	33.06	58.16
10	Canonical Correlation	0.76	0.71
11	Wilk’s Lamda	0.05	0.12
12	Significant p (<0.05)	0.00	0.00
13	Truss variable & Higher coefficient in Function	2_3: 309.52 1_5: 212.01	3_9: 582.49 4_8: (-)1034.82

### Discriminant analysis of principal components (DAPC)

**Figure 5:**
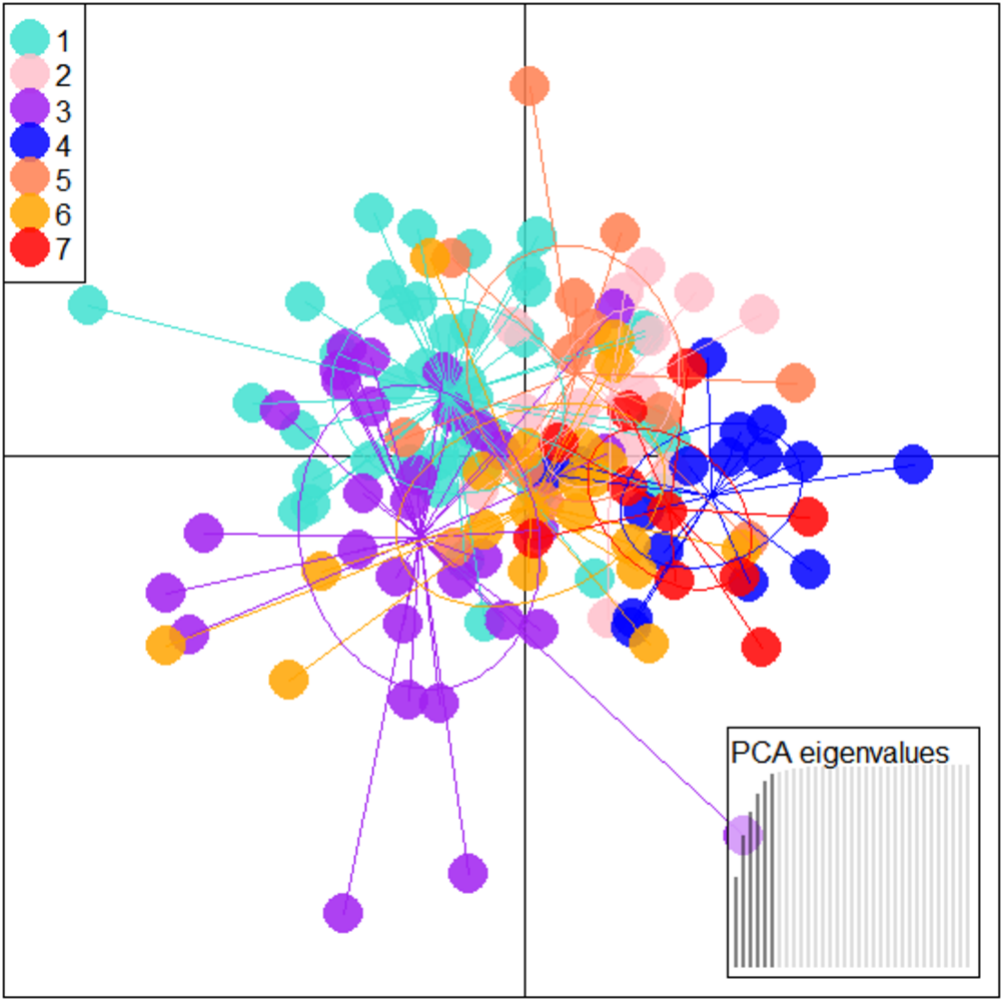
Specimen distribution over shape scores on PCA eigenvalues in DAPC analysis of data matrix 149 × 6PC. 1. Son; 2. Tons; 3. Ken; 4. Brahmaputra; 5. Ganga; 6. Gomti; 7. Gandak.

**Figure 6:**
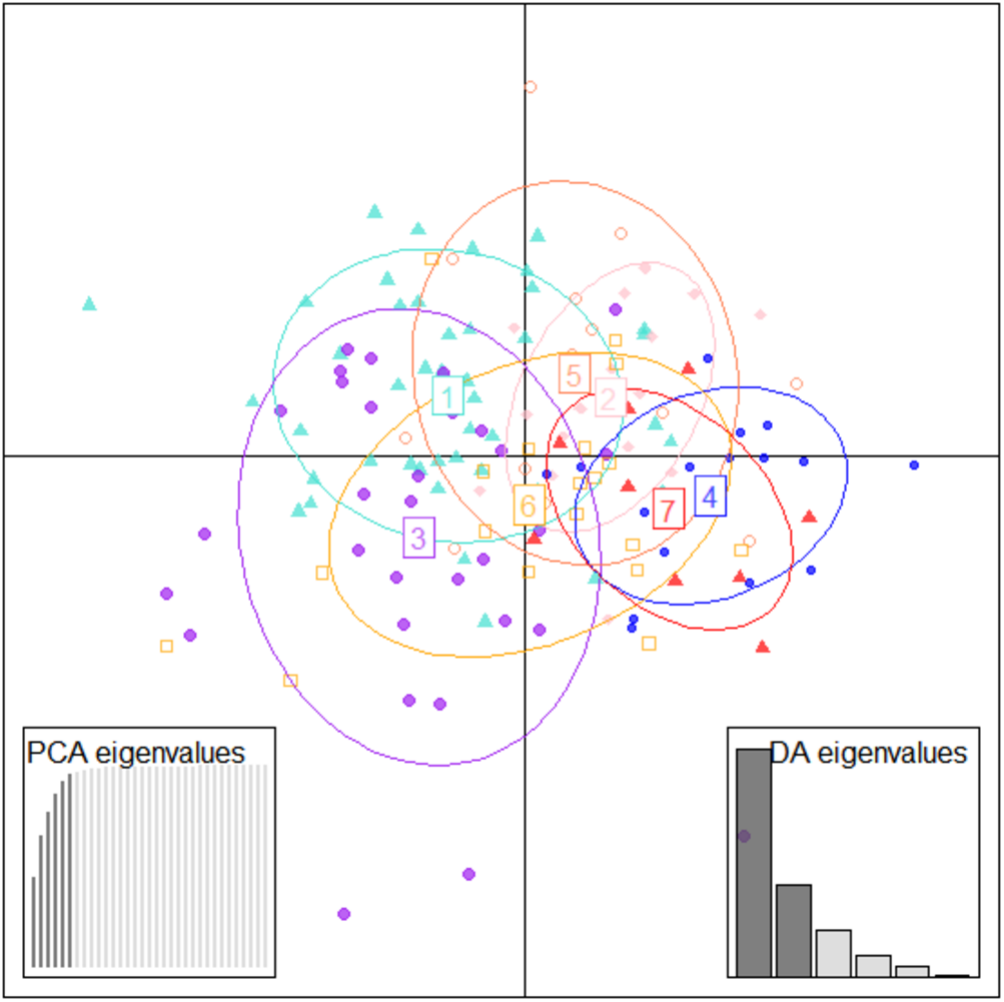
Specimen discrimination among seven locations over PCA & DA eigenvalues in DAPC analysis of data matrix 149 × 6PC. 1. Son; 2. Tons; 3. Ken; 4. Brahmaputra; 5. Ganga; 6. Gomti; 7. Gandak.

**Figure 7:**
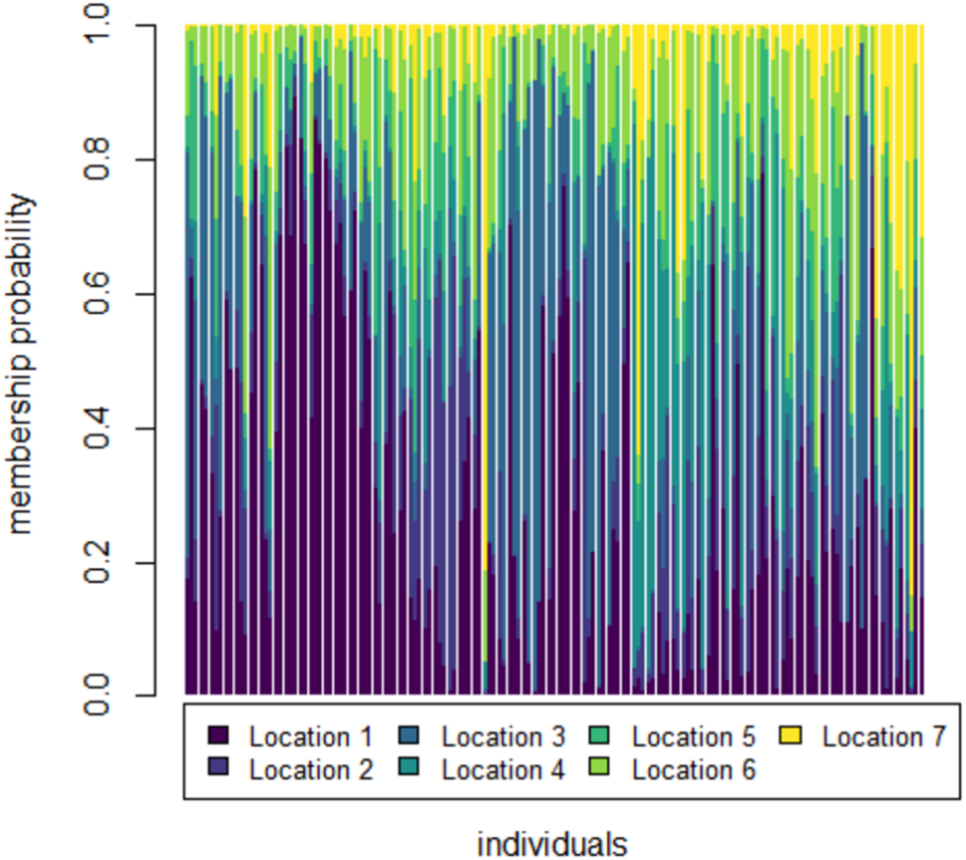
Membership probability distribution for specimen (individuals) among seven locations in DAPC analysis of data matrix 149 × 6PC. 1. Son; 2. Tons; 3. Ken; 4. Brahmaputra; 5. Ganga; 6. Gomti; 7. Gandak.

To further check for distinctness and similarity between the rivers DAPC was employed that provided five significant canonical functions that control 96.04% variation (Tables S1, S2). The discriminant scores for each river (centroid) was observed as different and PCA eigenvalue in DAPC analysis (Table 5, Fig. 5) that represents variations between locations and DAPC analysis of DA & PCA eigenvalues provides seven distinct locations (Fig. 6). Probability score-based membership of 149 specimens among seven locations has higher probability score for correct assignment for specimens from Gandak followed by Gomti and lowest for Son (Fig. 7).

**Table 5:** Group centroids evaluated using Functions (1, 2) of discriminant analysis of principal components (DAPC).

Sl No	Location	Functions (1, 2) evaluated at group centroids of locations	
		Function1	Function2
1	Son	−0.00	0.12
2	Tons	−0.86	0.11
3	Ken	0.37	0.09
4	Brahmaputra	−0.05	−0.08
5	Ganga	−0.87	−0.39
6	Gomti	0.33	0.16
7	Gandak	0.78	−0.64

### Comparison of performance of identified truss variables

**Figure 8:**
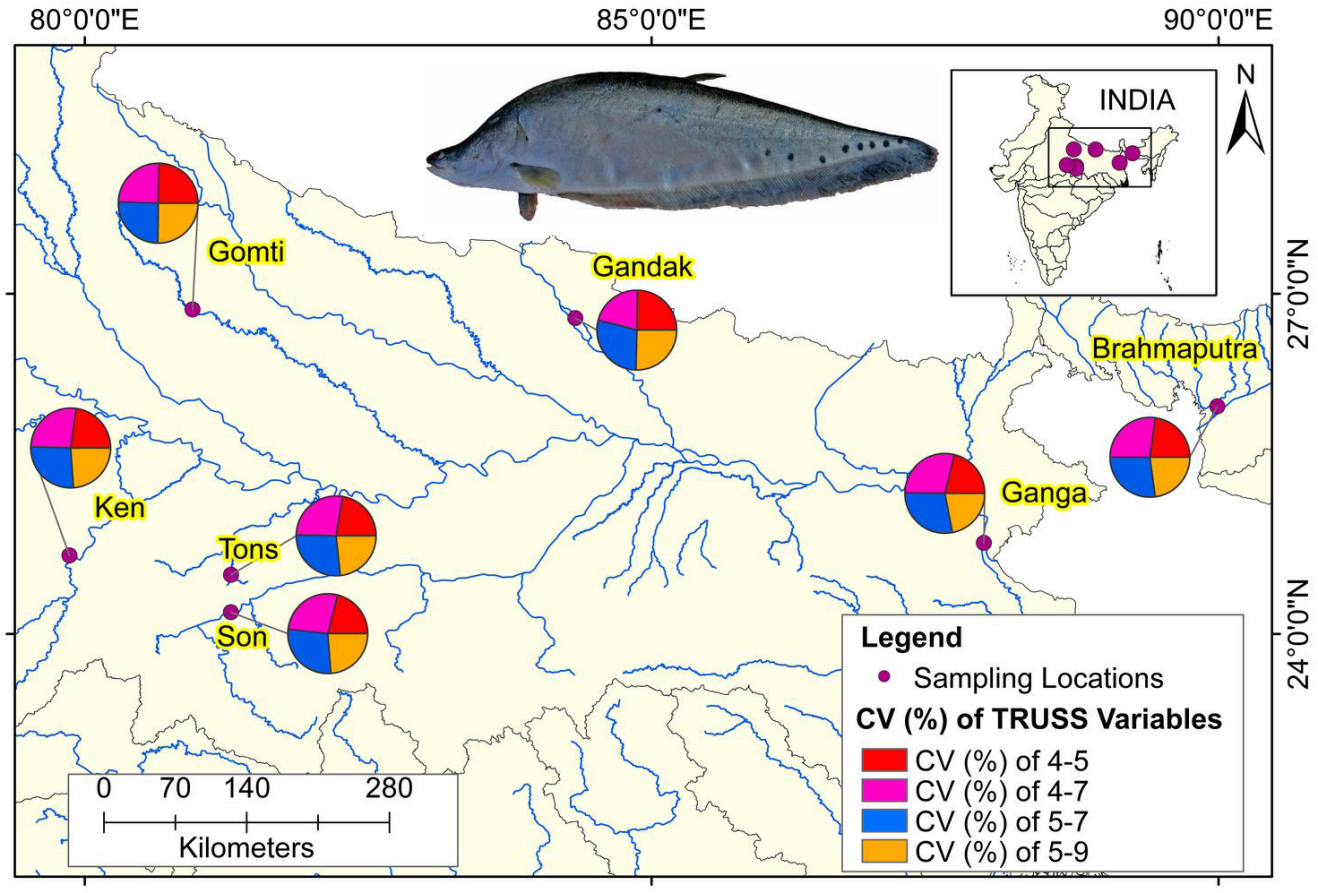
Ecomorphological distributions coefficient of variation on truss variables of specimens over locations. Map created using ArcGIS.

The comparison of performances of identified eight truss variables by principal components (Table 3) and from DAPC (Table 6) pointed out improvement in coefficient of truss variable in DAPC analysis that resulted into seven distinct centroids for seven rivers, under present study. This was further verified from the variation in performance of coefficients in PCA, CDFA and DAPC of truss variables 5_9 and 5_7, (identified through PCA with highest loadings: Figs. SA2–SAC). Comparative study of the three graphs indicated that truss variable (5_9 and 5_7) identified through PCA had higher loadings, but had little role with small coefficients in functions from CDFA, while had higher role as coefficients in functions from DAPC analysis of data. Higher coefficients of these two significant truss variables, 5_9 and 5_7 identified maximum distinctness of each river. Performance analysis of DAPC coefficients identified 5_9, 5_7, 4_7 and 4_5, as top truss variables, which distinguished specimens from different rivers (Table 3). The variation in coefficient of variance of these four identified variables between the rivers are depicted in Fig. 8 (Table S3).

**Table 6:** Performances of identified truss variable over principal component loadings, discriminant function (DF) coefficients and DAPC coefficients.

**Truss**	*PC loading of Truss		**CDFA coefficient of Truss**		**DAPC function coefficient of Truss**	
	**PC1**	**PC2**	**DFunction1**	**DFunction2**	**DAPC Function1**	**DAPC Function2**
5_9	0.90	0.33	–	–	−133.36	61.01
5_7	0.83	0.26	–	–	6.47	3.92
2_3	0.79	0.30	309.52	−199.39	0.15	0.73
1_5	0.75	0.58	212.01	37.52	0.23	−0.24
1_3	0.15	0.92	−157.30	−546.15	−0.01	0.03
3_8	0.44	0.78	–	–	1.53	3.77
4_7	−0.73	0.66	–	–	6.72	−6.97
4_5	−0.73	0.66	–	–	−3.64	5.53

**Notes.**

*Loading of Components significant (>or <0.13 or −0.13).

5_9: Distance between point perpendicular to dorsal fin origin and point perpendicular to posterior end of maxilla; 5_7: Distance between point perpendicular to dorsal fin origin and pectoral fin insertion; 2_3: Distance between posterior most aspect of neurocranium and dorsal fin origin; 1_5: Distance between anterior tip of snout at upper jaw and point perpendicular to dorsal fin origin; 1_3: Distance between anterior tip of snout at upper jaw and dorsal fin origin; 3_8: Distance between dorsal fin origin and posterior end of maxilla; 4_7: Distance between posterior end of vertebrae column and pectoral fin insertion; 4_5: Distance between posterior end of vertebrae column and point perpendicular to dorsal fin origin.

### Receiver operating characteristics (ROC) curve analysis

**Figure 9:**
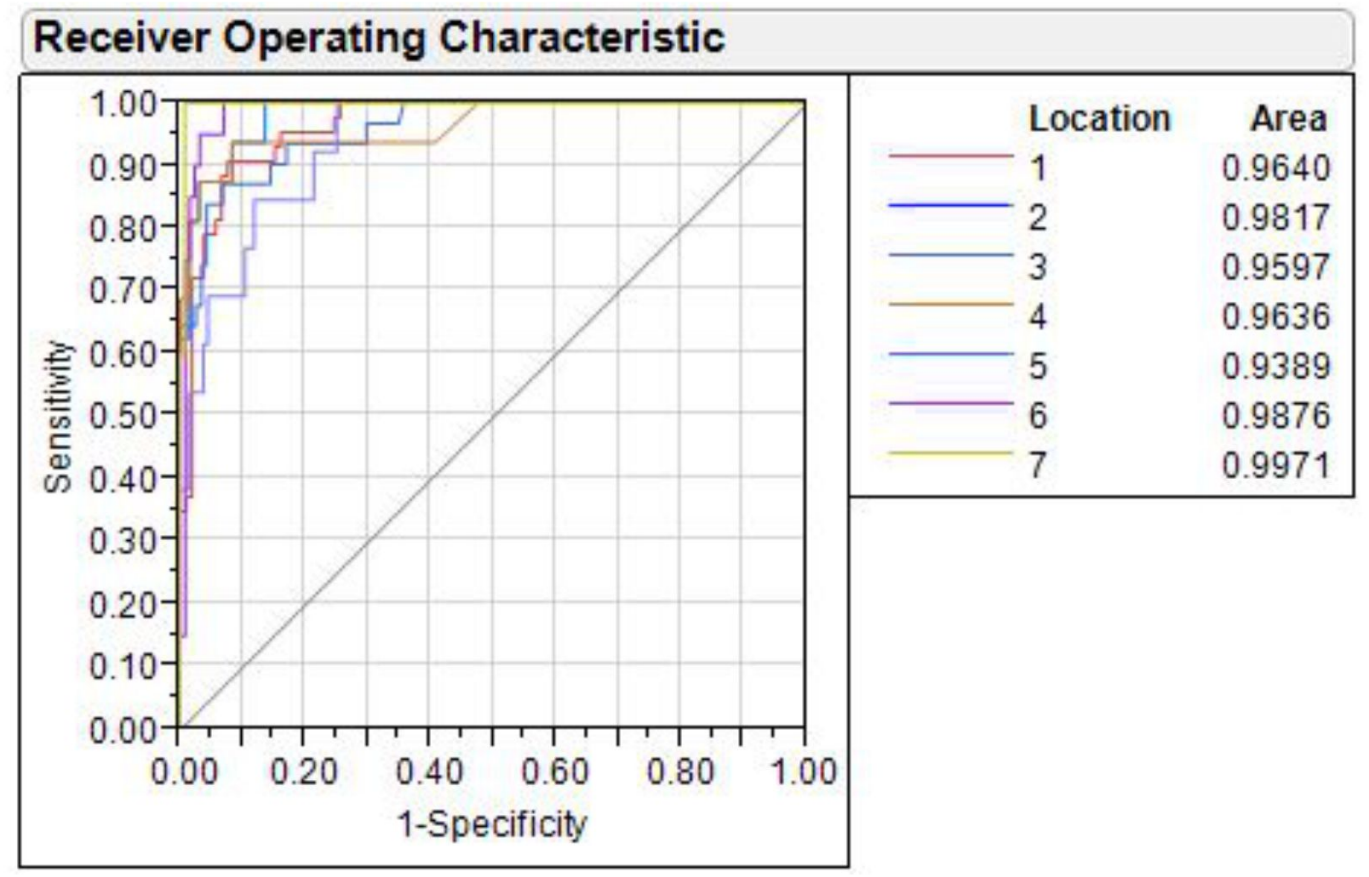
Receiver operating characteristic (ROC) curves for seven locations. 1. Son, 2. Tons, 3. Ken, 4. Brahmaputra, 5. Ganga, 6. Gomti, 7. Gandak.

The shape variation through ROC curve over (1-specificity) *vs.* sensitivity of discriminant function, analyzed for all seven rivers, showed that the value of area under curve (AUC) in each ROC ranged from lowest (0.9389) in Ganga to highest in Gandak (0.9971) (Fig. 9). AUC is a measure of sensitivity and as AUC values ranged between 0.90–1.00 for seven locations, it indicated an “outstanding performance” of identified truss variables in shape-based discrimination.

### Functional distribution of truss variables over seven rivers

**Figure 10:**
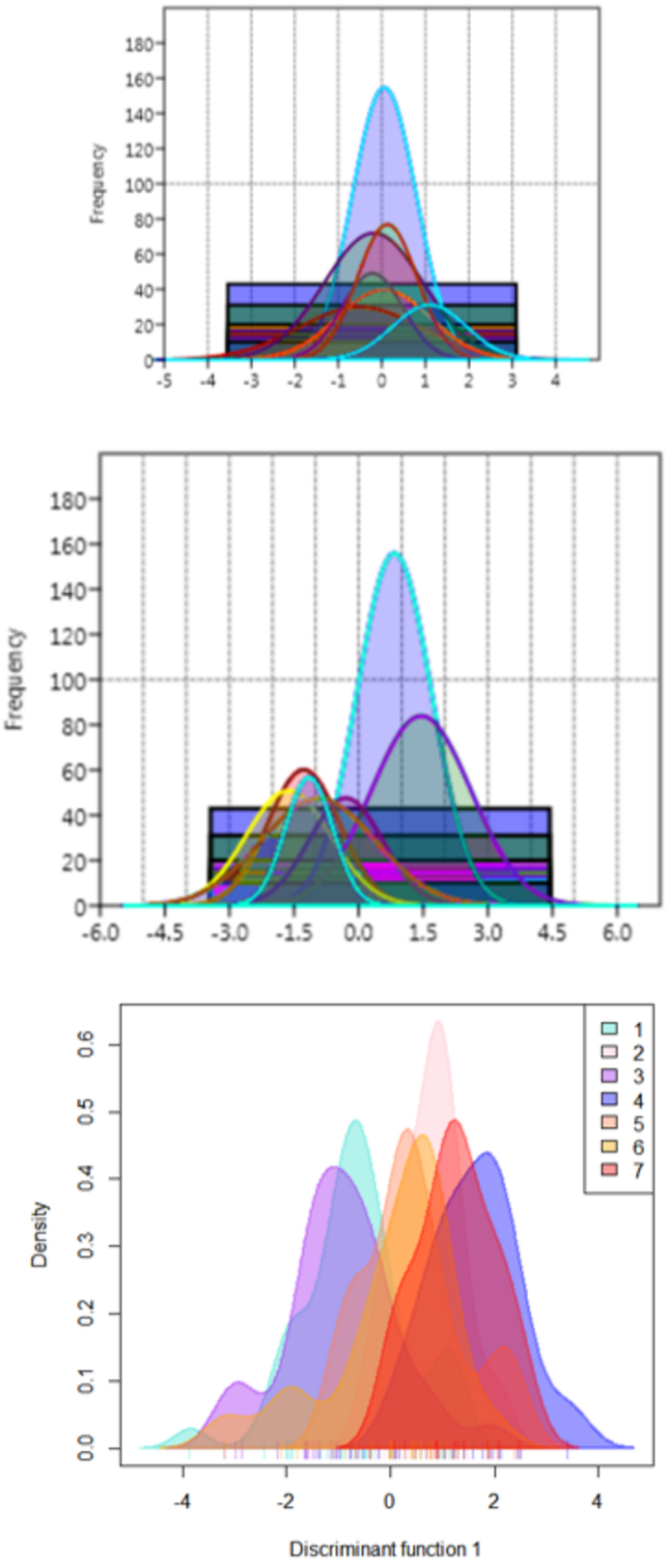
Seven location curves of specimen distribution over shape scores. (A) Principal component (PC1) from principal component analysis. (B) Canonical discriminant function (DAPC Function1), from DAPC analysisc. (C) Density-curves for specimen over canonical discriminant function (DAPC Function1), from DAPC analysis.

The functional distribution of the truss variables, which were identified from PCA and DAPC, when plotted to depict the shape variations over seven rivers, revealed that the distribution of PCA displayed seven graphs for seven rivers with similar position of peak (Fig. 10A), while from DAPC analysis, different and distinct distribution of specimens for seven rivers were observed (Fig. 10B). Thus, centroid of each location was dissimilar, though the observations were similar, indicating clear distinction and variation between locations (Fig. 10C).

### Thin-plate spline and warp density in shape analysis

**Figure 11:**
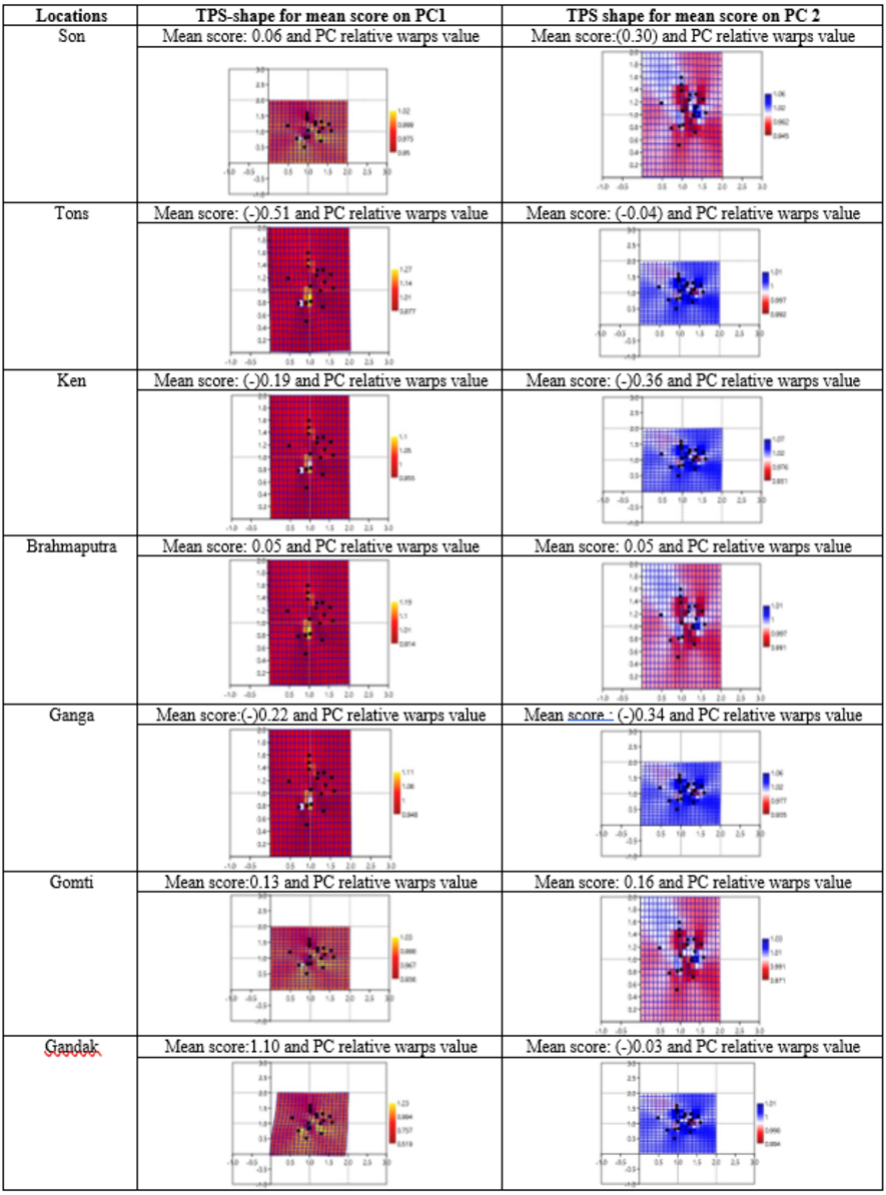
Thin plate splines and associated relative warps for shape variations over mean score on principal components (PC1, PC2).

**Figure 12:**
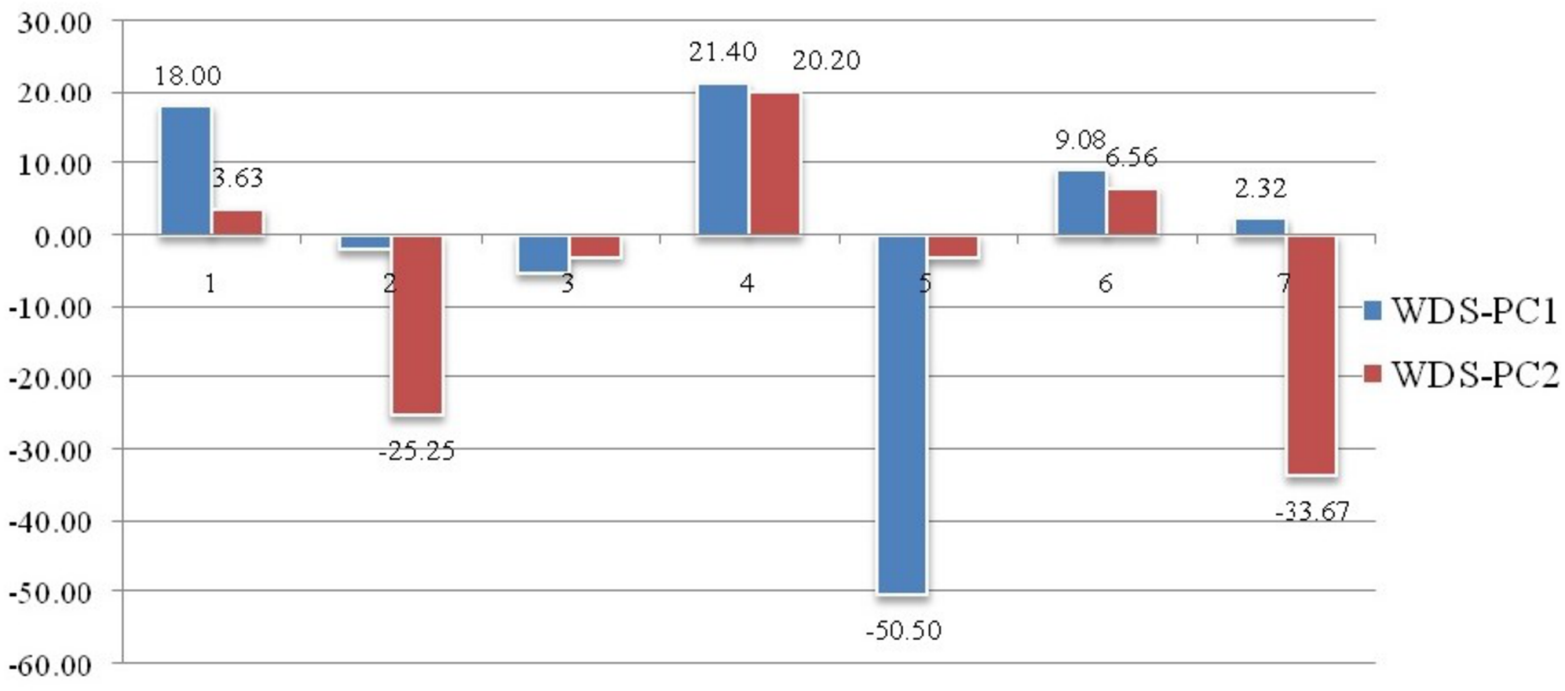
Shape variations through warp-density-score (WDS) over procrustes-principal components (PC1 & 2). 1. Son, 2. Tons, 3. Ken, 4. Brahmaputra, 5. Ganga, 6. Gomti, 7. Gandak.

Geometrical shape variation between the locations was gaged by evaluating various parameters under thin plate spline shape analysis. Maximum and minimum warp score from PCA on principal components; PC-1 was highest for Ken River (5.42) and least for Ganga (2.32) (Table 7). Similarly, the maximum and minimum warp score on PC-2 was highest for Gomti (1.56) and least for Ganga (1.15). Thin plate spline variation over both mean score and principal score ratio was assessed. The geometric shape (thin plate spline) variation, over mean score, through PCA (relative warps) revealed that the specimens from Tons and Gandak rivers had higher relative warps (deformation) as 1.27 and 1.23, respectively, whereas other river basins had relative warps (deformation) in the range of 1.01–1.06 (Table 7, Fig. 11). The thin plate spline shape analysis through PCA (relative warps PC1, PC2), over principal score ratio (PSR) on PC-1 denoted as TPS-PSR-PC1, indicated that relative warp was observed highest for Gandak and least for Tons (0.87) (Table 7). However, TPS-PSR-PC2 distinguished the seven rivers with scores in the range from 0.89–1.11 (Table 7, Fig. S3). As specimens from Gandak showed high relative warp over principal score ratio, it implies that the geometrical shape of these are clearly distinct from other locations. But there was some minor similarity in geometrical shape between locations *viz.,* Son and Brahmaputra on preliminary basis (Fig. 12). Thus, to capture small-scale deformations and variations between locations, warp density scores on principal components (PC1 and 2) was also assessed. The warp-density-score (WDS) for seven rivers over PC1 had values in range from (-) 50.50 to 21.40 while WDS values scores over PC2 ranged from (-) 33.67 to 20.20 (Table S4). The WDS over principal components (PC 1, 2) indicated the extent of shape variation of specimens collected from seven rivers (Table S4, Fig. 12). WDS indicated clear score for all locations indicating distinction between the specimens over locations shows shape-based variations.

**Table 7:** The distribution of mean, standard deviation (SD), minimum, maximum and range on scores over principal component PC1 & 2 along with thin plate spline (TPS) through PCA (relative warps) analysis.

**Rivers**	**Mean, SD, minimum, maximum and mean score linked PCA (relative warps) for specimens of locations on Principal component PC1(PC2)**					**Thin plate spline (TPS) - PCA (relative warps PC 1, 2)**			
						**Principal score ratio (PSR) of TPS image of specimens over PC1&2**		**Principal score Ratio over PC1, PC2**	
	**Mean**	**SD**	**Min**	**Max**	**Mean score linked PCA (relative warps)**	**PC1** **Max** **(min)**	**PC2** **Max** **(min)**	**TPS-PSR-PC1**	**TPS-PSR-PC2**
Son	0.06 (0.30)	0.73 (1.04)	−2.70 (−2.69)	1.48 (2.40)	1.01 (1.06)	3.09 (2.70)	1.53 (1.64)	1.14	0.93
Tons	−0.51 (−0.04)	1.41 (1.11)	−3.54 (−2.26)	1.65 (1.49 )	1.27 (1.01)	3.34 (3.82)	1.37 (1.54)	0.87	0.89
Ken	−0.19 (−0.36)	1.14 (1.08)	−2.15 (−2.19)	3.09 (1.73)	1.10 (1.01)	5.42 (1.96)	1.42 (1.52)	2.77	0.93
Brahmaputra	0.05 (0.05)	1.06 (0.97)	−1.64 (−1.33)	1.79 (2.20)	1.01 (1.01)	3.54 (1.27)	1.50 (1.32)	2.79	1.14
Ganga	−0.22 (−0.34)	0.70 (0.56)	−1.65 (−1.17)	0.94 (0.52)	1.11 (1.06)	2.32 (1.28)	1.15 (1.28)	1.81	0.90
Gomti	0.13 (0.16)	0.69 (0.97)	−1.27 (−1.69)	1.43 (2.63)	1.03 (1.03)	3.02 (1.06)	1.56 (1.40)	2.85	1.11
Gandak	1.10 (−0.03)	0.85 (0.59)	−0.57 (−0.74)	2.46 (1.00)	1.23 (1.01)	4.50 (1.03)	1.27 (1.18)	4.37	1.08

### Procrustes geometric morphometric analysis

**Figure 13:**
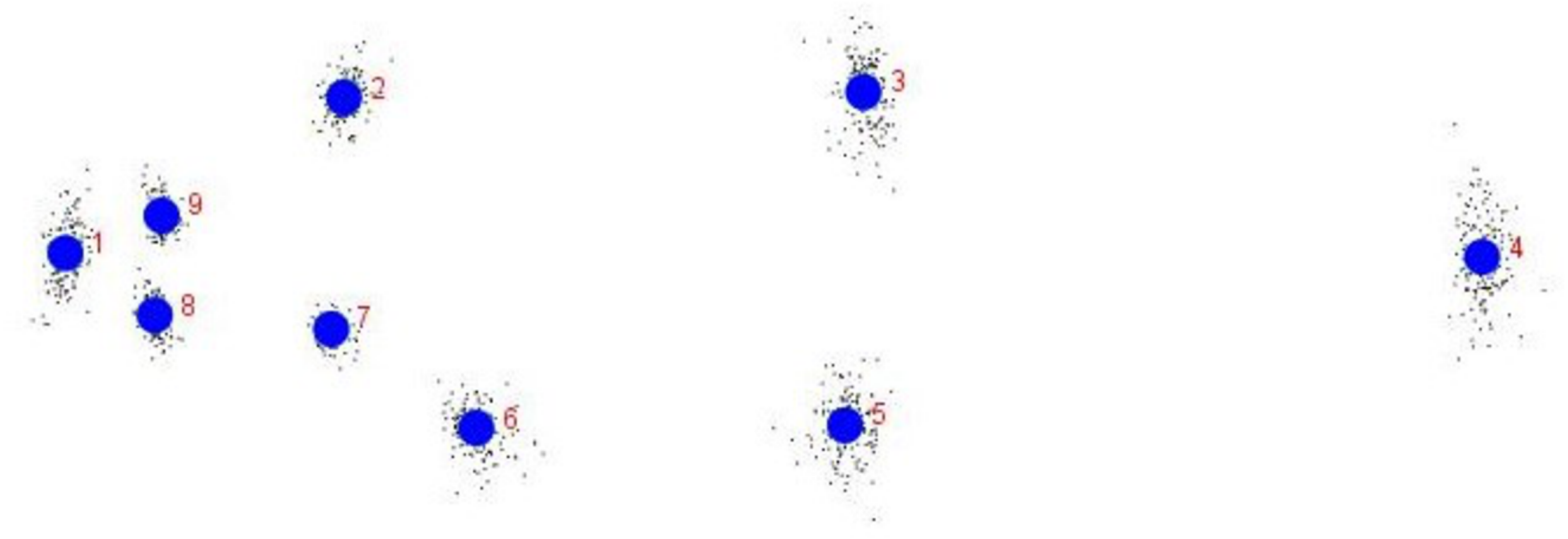
Generalised Procrustes shape (GPS) over nine landmarks of 149 specimens from procrustes geometric morphometric analysis.

**Figure 14:**
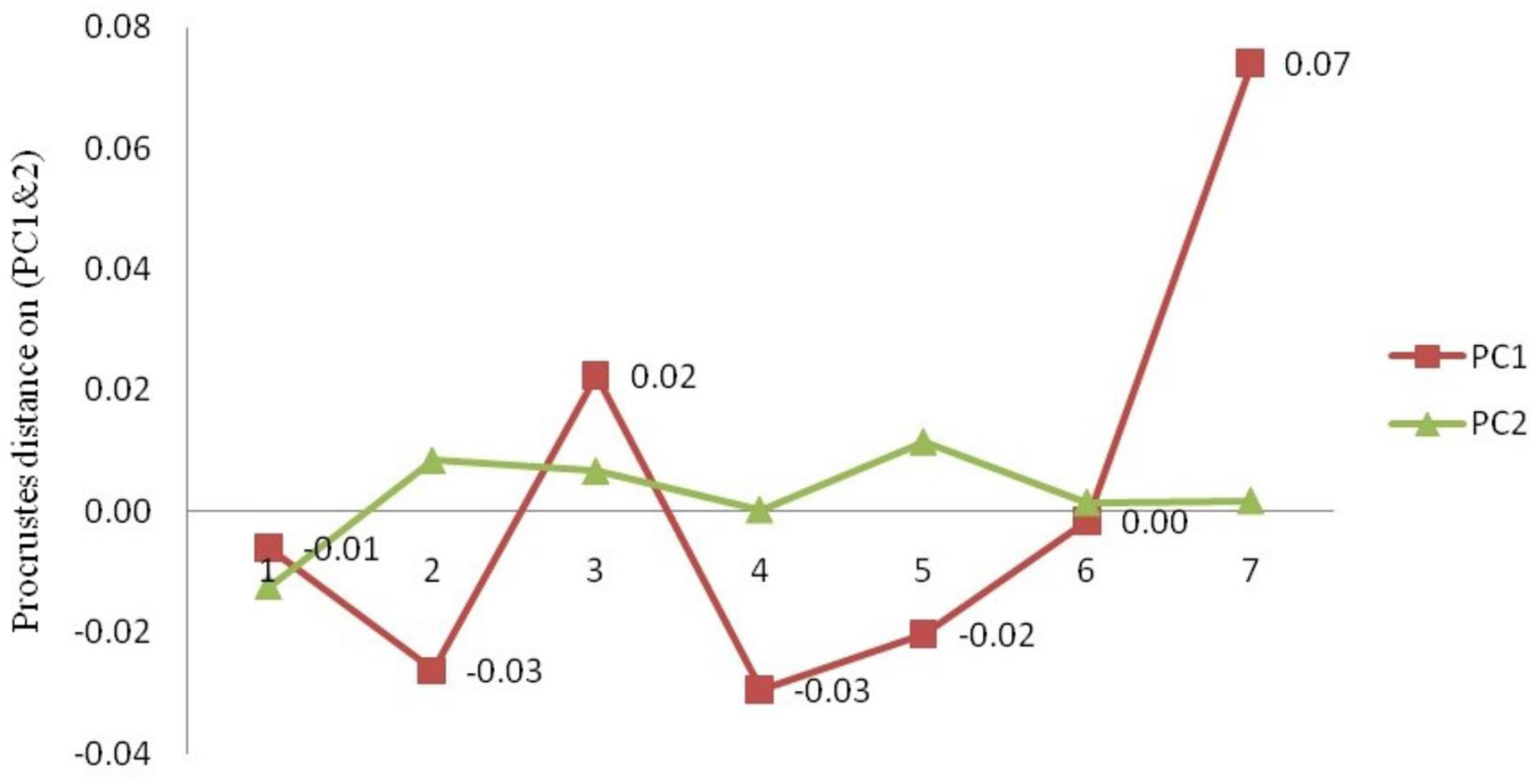
The mean procrustes distance on procrustes-principal components (PC1& 2) from landmark-PCA (relative warps) deformations. 1. Son; 2. Tons; 3. Ken; 4. Brahmaputra; 5. Ganga; 6. Gomti; 7. Gandak.

The procrustes analysis of variance (ANOVA) of all specimens over the nine landmark-coordinates, to determine centroid size & shape variation (Tables S5, S6) indicated that there existed significant (*p* < 0.05) centroid size-based differences and significant procrustes shape variations for fish specimens from seven rivers. Highest procrustes distance (highest variation w.r.t. mean shape within location) was observed for Gandak (0.07), followed by Ken (0.02), Gomti (0.00), Son. (−0.01), Ganga (−0.02) and the least for Tons and Brahmaputra (−0.03) (Figs. 13 and 14) from generalized procrustes shape. This indicates that maximum distinct shape from generalized procrustes shape was observed in Gandak specimens while it was least for Tons and Brahmaputra.

### Generalised procrustes shape variations over principal components analysis and between locations

**Figure 15:**
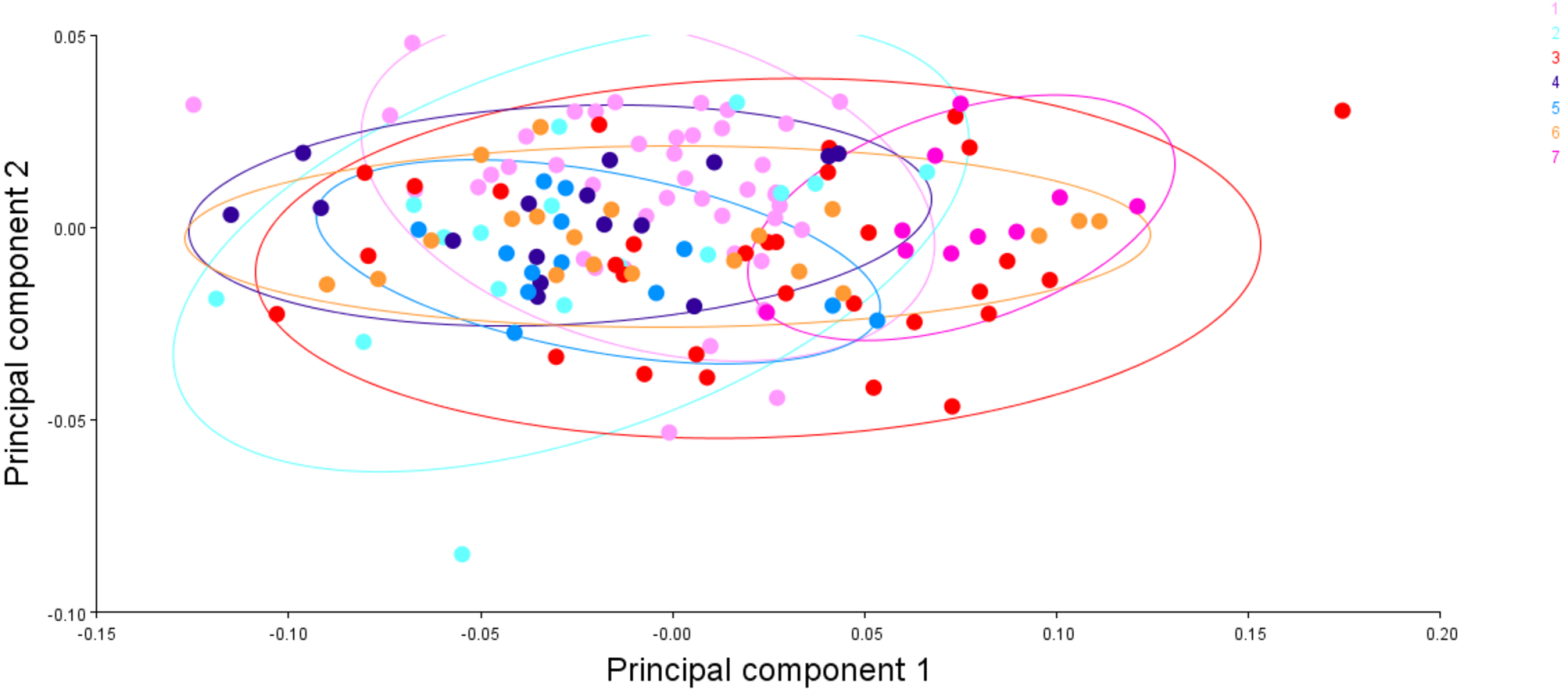
The generalised procrustes shape distribution of 149 fish specimens from seven locations, confidence ellipses with probability 0.95, over procrustes principal components (PC1,2). 1. Son; 2. Tons; 3. Ken; 4. Brahmaputra; 5. Ganga; 6. Gomti; 7. Gandak.

To visualise the shape variation, landmark-coordinate data matrix was subjected to principal component analysis that identified seven principal components, which controlled 97.32% of total variance. The shape variation of 149 fish specimens has been observed on maximum & minimum scores on procrustes-principal components PC1 (PC2) (Table S7, S8, Fig. 15). The differences in mean shape over procrustes distances were significant for Tons & Brahmaputra (procrustes distance: 0.01903507, Mahalanobis distance: 3.9625, *p* = 0.0011) and between Gandak & Gomti (procrustes distance: 0.08225527, Mahalanobis distance: 5.2176, *p* = 0.003). The shape variations for location Tons & Brahmaputra was also observed over shape differences through lollipop graph, discriminant scores graph and cross-validation graph. Similar graphical study was also performed for locations; Gandak & Gomti (Fig. S4). It was noted that two locations (Tons & Brahmaputra) though have shape differentiation through lollipop graph (Fig. S5), does not indicate much shape differentiation over landmarks as the tail length at all landmarks were minimal while significant procrustes distance proves shapes differentiation. Similarly, procrustes distance was recorded as 0.08225527, while, Mahalanobis distance was found to be 5.2176 at *p*-value 0.0003 between Gandak and Gomti indicating significant shape distinction. Cross validation and shape differentiating lollipop graph indicates clear differentiation in this case between locations as the tail length at almost all landmarks is high (Figs. S6A, S6B).

### Procrustes Shape Modularity over subset of landmarks

A set of nine landmarks identified were partitioned into two subsets to check for shape variation through procrustes shape modularity. Landmarks 1, 8, and 9 formed a subset that were related to body feature; hump, and landmarks 4, 5, and 6 formed another subset related to fins. The shape modularity hypothesis of landmark subsets (Figs. S6A, S6B) indicated that both the subsets identified had higher relative variance coefficient (0.85, 0.85) (Table S9) which indicated the significant role of landmarks in body shape variations (Figs. S7A, S7B). The utility of procrustes shape modularity in shape-based clustering of specimens over subset of landmarks (1,8,9 *vs.* 2,3,4,5,6,7) and subset of landmarks (4,5,6 *vs.* 1,2,3,7,8,9.) with the clustering criteria as relative variance (RV) coefficients greater or less than 0.85 was carried out (Figs. S8A, S8B).

## Discussion

In current investigation, geometric morphometric analysis was employed to quantify the distribution and pattern of intra-specific phenotype diversity in notopterid fish, *C. chitala* from different rivers. Nine landmarks (36 truss variables), covering the entire shape of fish body, were used and subjected to multivariate analysis. The truss-based shape morphometry relies on stability of principal components, which is largely impacted by sample size and optimum N (sample size) to P (No. of truss elements) ratio recommended in the range of 3.0–8.0 (Kocovsky, Adams & Bronte, 2009). The N to P ratio in this study confirmed to this optimum recommendation.

The results suggest comparatively higher resolving power of DAPC over CDFA for capturing shape variability and location discrimination. DAPC identified seven distinct centroids for the specimens from each geographic location and thus, clearly deciphered the phenotype diversity in *C. chitala* wild populations. Similar results were also observed in bent-toed geckos (Kaatz, Grismer & Grismer, 2021) and water frogs (Papežík et al., 2021). The inference from present investigation also suggested use of log morphometric data, as an improvement over commonly used mtrans data and is also concordant to our earlier results on a carp species, *Systomus sarana* (Biswal et al., 2020). Superiority of log morphometric data (over mtrans) is judged from the higher standard deviation and larger range values as compared to that obtained from mtrans data. The mtrans data obtained after applying transformation as proposed by Elliott, Haskard & Koslow (1995), may have influence of conflation of intra- with interspecific variation of fish specimens from different locations (Reiss & Schmid-Araya, 2010; Kaatz, Grismer & Grismer, 2021). The location with ‘AUC’ value in range from 0.90–1.0 and 0.80–0.90 is assessed as outstanding and excellent performance respectively for shape analysis through discriminant functions (Mandrekar, 2010). This study revealed that the truss variables associated with fins and hump region are the important contributors to the shape variation. Sarkar, Deepak & Lakra (2009) had noticed the phenotypic plasticity in fin lengths of *C. chitala,* collected from various locations. Morphological variations, linked to locomotion, were also reported in fishes, such as *Astatotilapia burtoni* (Theis et al., 2014) and *Lepomis cyanellus* (Gaston & Lauer, 2014) and *Systomus sarana* (Biswal et al., 2020). Notopterids are classified as body-caudal-fin swimmers (BCF) and have gymnotiform mode of swimming (Blake, 1983a). This is characterized by the ribbon-like motion of elongate ventral/anal fin, while the body remains relatively stiff (Akanyeti et al., 2017). Such swimming is facilitated by their lean bodies and reduced dorsal fins and is also observed in *C. chitala* (Whitlow, Santini & Oufiero, 2019). *C. chitala* swim in the sub-carangiform mode (Webb, 1975; Blake, 1983b), where the caudal fin undergoes large movements that generate substantial side forces or recoil forces (Lighthill, 1970). Increased body depth and reduced cross-sectional depth posteriorly (narrow-necking) reduces the influence of recoil forces. Notopterids exhibit these morphological adaptations which help to minimise recoil forces. Similarly, another notopterid, *C. ornata* employs pectoral fin for steady swimming while always revert back to body undulations for accelerating forward (Akanyeti et al., 2017).

The environment and water flow determine the propulsive motion of fins. The mechanisms used to explain the hydromechanics of fin propulsions are related to momentum in fluid (Blake, 1980), resistive forces developed on fish body (Lighthill, 1975) or reactive (inertial) force developed in fluid by swimming fish (Lighthill, 1971). Of these, reactive force developed in fluid by undulating movements of body and fins is explained for the Notopterids. This theory is guided with three basic assumptions that the length of the fin does not change, fin depth is also either constant or varies smoothly by a small amount over the body length and each vertical water slice perpendicular to the motion of any given part of the fin is influenced mainly by neighbouring parts of the fin close to that slice (Blake, 1983c). In *C. chitala*, the first two assumptions are clearly met, however there always occurs changes in the environment *i.e.,* vertical water, due to depth and water pressure, causing changes in the kinematics of fin. Thus, the variation in the hydrodynamics of the vertical water affects the kinematics of fins and consequently leading to adaptation during the evolutionary process and possibly is responsible for phenotypic variation in *C. chitala,* collected from different rivers.

Landmarks associated with hump were also found to play key role in morphological variation between locations. These observations are similar to the observation of Sarkar, Deepak & Lakra (2009), who had observed distinct variation based on characters associated with fins and the hump of *C. chitala.* Truss variables (5_9 and 5_7), based on their higher loadings on principal component analysis, showed their potential for phenotypic differentiation among individuals collected from different rivers in *C. chitala*. The ROC analysis, considered for diagnostic test performance, confirmed the extent of shape variation. The maximum shape variation was observed in the river Gandak, compared to other rivers, based on higher values of relative warp over both mean score and principal score ratio. This was also supported by maximum procrustes distance that indicated clear distinction between the specimens of *C. chitala* over different locations. Results of thin plate splines (Bookstein, 1989; Bookstein, 1991), showed similarity in geometrical shape between rivers, Son and Brahmaputra, however warp density scores (WDS) clearly indicated variation between them. WDS indicated distinct scores for all locations, which indicated shape-based variation between locations.

The phenotypic divergence has been linked to geographic isolation, restriction in genetic flow and varying habitat conditions (Hanif et al., 2019). Isolated populations of fish species respond to micro-evolutionary processes and are likely to undergo phenotypic alterations (Chen, Wu & He, 2018). In *C. chitala,* majority of the truss variables showed similarity between locations, indicating existence of ancestral phenotype characters, which might have carried through generations of fragmentation from the common ancestor. The rivers studied in this study, have different hydrological flows, undergone vicariant events and have different time of origin in historical past. The rivers Son, Tons and Ken, which flow to the southern side of the Ganges, have been in existence (Gondwana Land) before the Himalayas originated leading to the formation of rivers like Ganga, Gandak and altered flow of Brahmaputra. Tons river catchment area exhibits an intricate pattern of mountain system consisting of high mountains (Krishan, Kushwaha & Velmurugan, 2009) while Ken River is affected by construction of a number of irrigation projects, which might have had an adverse impact on physicochemical, biological, and fishery parameters (Joshi et al., 2017) and is flanked by undulating plateau with sandstone, shale and limestone. River Son, a southern tributary of Ganga River, is also known to possess many dams, reservoir and hydropower plants, as it is a seasonal river, all of which might have had an influence on the environmental parameters. River Gandak is a flood prone river, where studies have revealed a lower diversity and declined fish catch owing to siltation and changes in land use pattern, over the years (Shrivastava, 2013). Gandak river basin has diverse geological record ranging from cretaceous-tertiary igneous and metamorphic rock followed by Precambrian rocks to quaternary sediments (Chaubey et al., 2021). Thus, there exists predominant variations/differences in the habitat features and environmental parameters between locations with reference to the rocks, minerals, terrain, water current and water level. The present phenotypic differentiation in *C. chitala* might be due to varying habitat conditions particularly, temperature, salinity, turbidity, and alkalinity (Kristjansson, 2005; Hanif et al., 2019; Biswal et al., 2020). The present study, clearly differentiated phenotype variants of *C. chitala*, which are likely to have the control of genetic makeup, evolved in response to the adaptive needs to suit changing environment, after fragmentation of the populations. It is likely that these could be “phenotypic stocks, that have adapted separately to their respective environments” (Coyle, 1997). Though, the genetic analysis presented insignificant divergence between Ganga and Brahmaputra (Dutta et al., 2020), phenotypic investigation displayed, the samples drawn from these two rivers, as separate centroids. Thus, the significant phenotypic plasticity could discover at least seven morphotypes of *C. chitala* in the subpopulations found in different rivers, which may be due to diverse habitats as explained above. However, use of advanced variable molecular markers in combination with phenotyping tools may unravel the obscure diversity in *C. chitala*, a species of importance from evolution and conservation point of view. Low genetic differentiation, determined in earlier studies through molecular markers, has been considered as the characteristic of genus *C. chitala* (Dutta et al., 2020). Mandal et al. (2012) attributed this to possible reduction of population during historical desiccation periods and reported in other primitive animals such as lungfishes, snub-nosed monkey etc. The reports of discord among the genetic differentiation revealed through genotype and phenotype data are not uncommon. One of the important reasons can be the inadequate part of genome analysed through direct assessment by molecular markers in the species with low differentiation. Kumar et al. (2006) could not find differences among the phenotypically established breeds of riverine buffalos through use of 27 SSR markers. Similarly, Alves et al. (2013) observed that the molecular markers used might not be capturing the portion of genome which controls phenotype differences, while working on accessions of Brazilian physic nut through joint use of molecular and phenotypic tools. It will be important to understand intraspecific differentiation in this primitive species, through use of highly polymorphic nuclear markers, including genome-wide markers, for finding fine-scale divergences in the native distribution of wild *C. chitala*. The genotype and phenotype are important for conservation, evolution, phylogeography and aquaculture breeding programs (Liao et al., 2021). The present results emphasize the positive role of phenotyping tools in determining population differentiation in native distribution range of fish species in the wild and significant distinguishing characters. Though, it may not be always possible to directly link to the part of genome controlling the phenotype differences, nevertheless, these can be useful tools yielding genetic divergence data complementary to molecular markers analysis.

## Conclusion

Our present investigation identified phenotypic variance in *C. chitala,* which was supported by various statistical tools. The present study proposes a tool kit for this purpose, through use of truss landmarks covering the whole body, utilizing log-transformed data employing DAPC to explore differences between locations and simultaneously minimising differences within locations, thin plate splines and procrustes analysis for body-shape visualisation. These phenotyping tools have great importance for the scientific management and conservation of a species. The results of this investigation indicate that molecular markers were found limited in finding significant distinction in *C. chitala* from Ganga and Brahmaputra river basins. However, the species exhibited significant phenotypic plasticity which explicitly indicates the presence of at-least seven different morphotypes of *C. chitala* in the subpopulations found in different rivers. Use of advanced variable molecular markers in combination with phenotyping tools is highly desirable in future, for documenting diversity in this *C. chitala,* a species of importance from evolution, conservation and aquaculture point of view.

##  Supplemental Information

10.7717/peerj.13290/supp-1 Supplemental Information 1 Sampling locations of *Chitala chitala* from Indian rivers Map created using ArcGIS.

10.7717/peerj.13290/supp-2 Supplemental Information 2 Truss performance loadings on Principal components from a. PCA b. CDFA c. DAPC

10.7717/peerj.13290/supp-3 Supplemental Information 3 Thin plate spline (TPS) principal score ratio (PSR) analysis on maximum & minimum score over principal components 1. Son 2. Tons, 3. Ken 4. Brahmaputra 5. Ganga 6. Gomti 7. Gandak

10.7717/peerj.13290/supp-4 Supplemental Information 4 Cross-validation scores & Procrustes shape difference for locations Tons (2) and Brahmaputra (4)

10.7717/peerj.13290/supp-5 Supplemental Information 5 Cross-validation scores & procrustes shape difference (lollipop graph) for locations; Gomti (6) and Gandak (7)

10.7717/peerj.13290/supp-6 Supplemental Information 6 Partitioning of nine landmarks into subset 1, highlighting the hump (Blue) b. subset 2, highlighting the ventral fins (blue)

10.7717/peerj.13290/supp-7 Supplemental Information 7 A. Relative variance (RV) coefficient as co-variation measure of subset one, highlighting the hump b. subset two, highlighting the ventral fins

10.7717/peerj.13290/supp-8 Supplemental Information 8 Distribution of specimen with relative variance of landmarks of a. hump region b. ventral region in relation to relative variance value (0.85) 1. Son 2. Tons, 3. Ken 4. Brahmaputra 5. Ganga 6. Gomti 7. Gandak

10.7717/peerj.13290/supp-9 Supplemental Information 9 Wilks’ Lamda in testing of functions derived from discriminant analysis on principal components (DAPC)

10.7717/peerj.13290/supp-10 Supplemental Information 10 Eigenvalues of functions derived from discriminant analysis on principal components (DAPC)

10.7717/peerj.13290/supp-11 Supplemental Information 11 Truss variables-shape variation on Coefficient of Variation (CV %) analysis across rivers

10.7717/peerj.13290/supp-12 Supplemental Information 12 Warp density score (WDS) of shape over principal components (PC1, PC2) for shape-based variations

10.7717/peerj.13290/supp-13 Supplemental Information 13 Procrustes ANOVA for centroid size-based differences

10.7717/peerj.13290/supp-14 Supplemental Information 14 Procrustes ANOVA for shape-based differences

10.7717/peerj.13290/supp-15 Supplemental Information 15 Generalized Procrustes shape variation associated with principal components from principal component analysis

10.7717/peerj.13290/supp-16 Supplemental Information 16 Procrustes distance (mean value) on principal components (PC-1&2) through PCA (relative warps) analysis

10.7717/peerj.13290/supp-17 Supplemental Information 17 The landmarks over body of fish specimen partitioned into two subset with relative variance (RV) coefficient

10.7717/peerj.13290/supp-18 Supplemental Information 18 C. chitala landmark coordinate raw data

10.7717/peerj.13290/supp-19 Supplemental Information 19 C. chitala truss measurements raw data

## Additional Information and Declarations

### Data Availability

The following information was supplied regarding data availability: The morphometric measurements of the fish species recorded from various river basins of India are available in the Supplemental File.
