# Supplementary figures and images for: Phenotypic variation of Chitala chitala (Hamilton, 1822) from Indian rivers using truss network and geometric morphometrics
Source: PeerJ. 2022 Apr 18;10:e13290. doi: 10.7717/peerj.13290 (PMC9022642; doi:10.7717/peerj.13290)

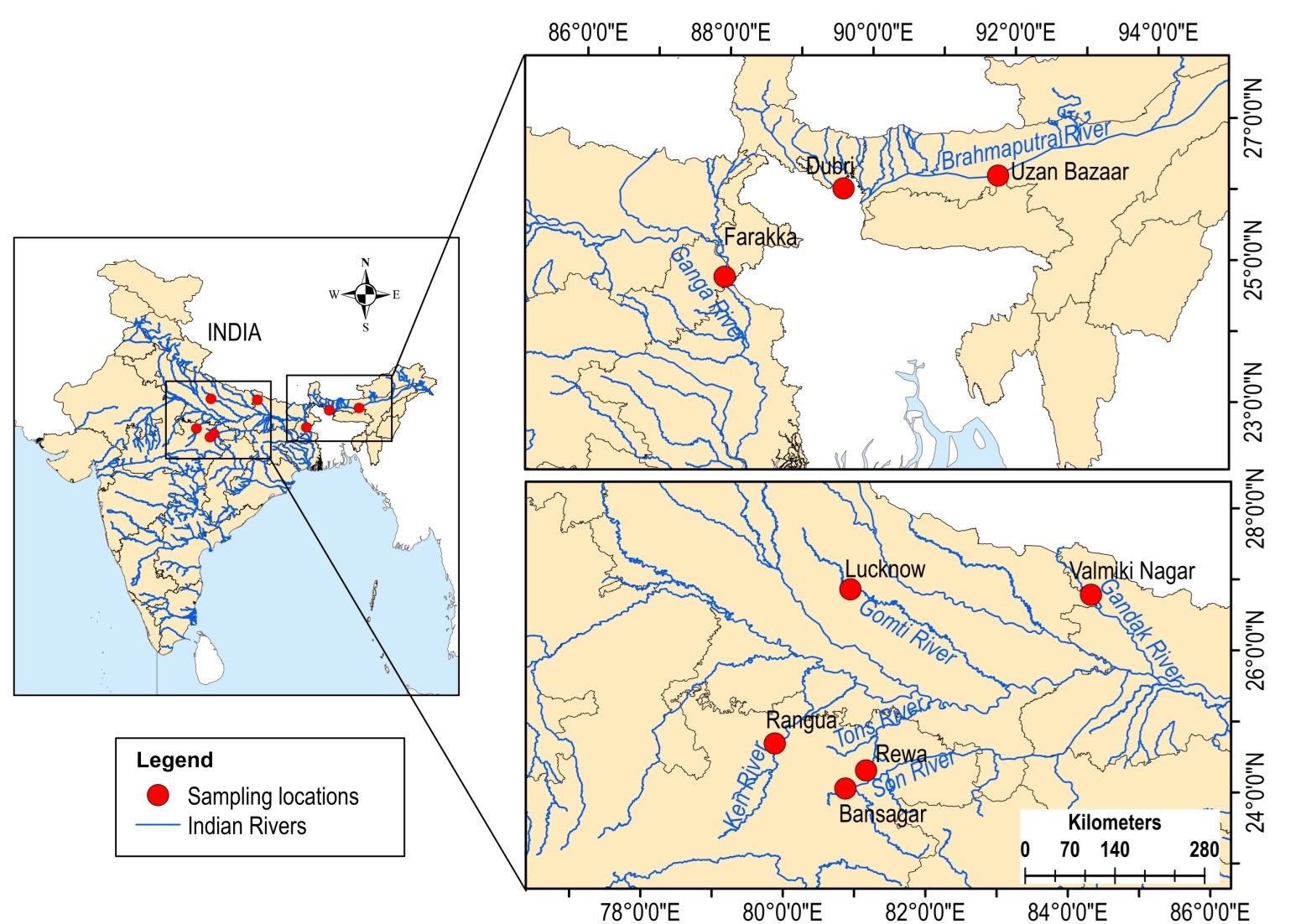

Supplement: Supplemental Information 1 — Map created using ArcGIS. [file peerj-10-13290-s001.jpg]

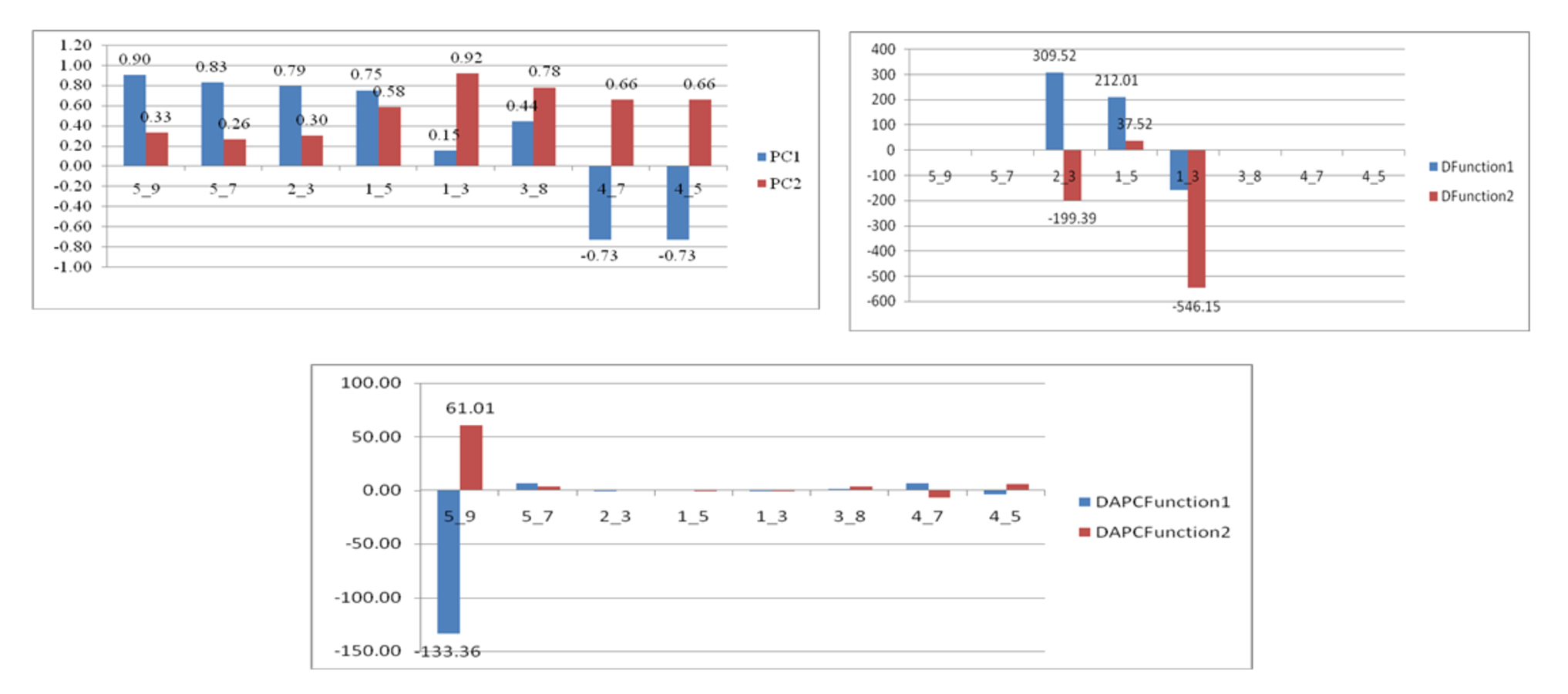

Supplement: Supplemental Information 2 [file peerj-10-13290-s002.png]

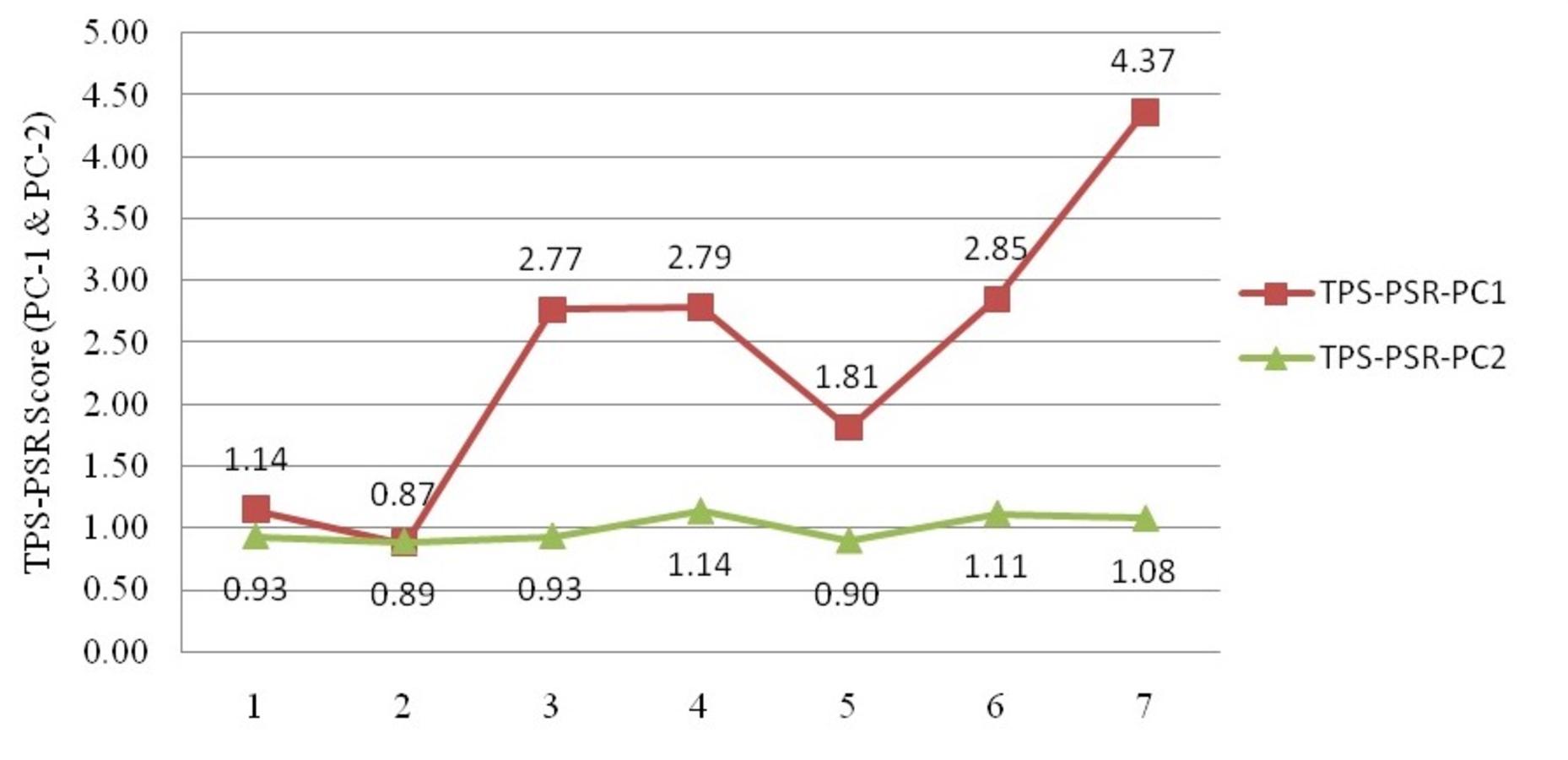

Supplement: Supplemental Information 3 — 1. Son 2. Tons, 3. Ken 4. Brahmaputra 5. Ganga 6. Gomti 7. Gandak [file peerj-10-13290-s003.jpg]

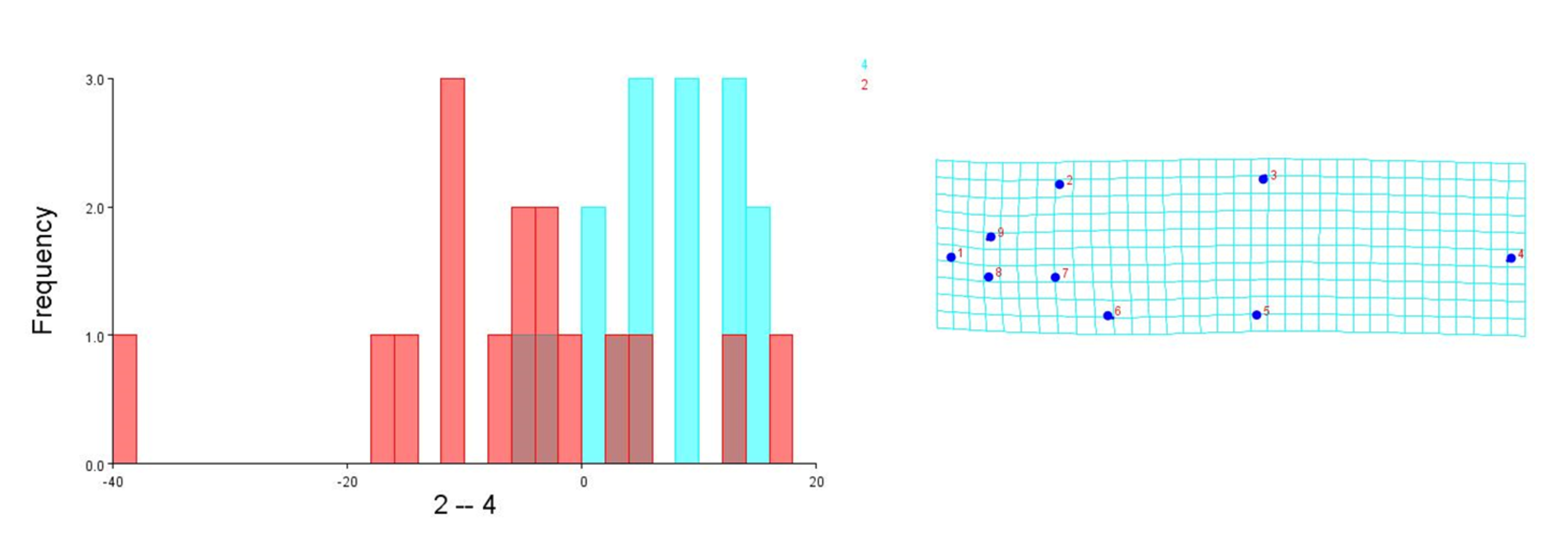

Supplement: Supplemental Information 4 [file peerj-10-13290-s004.png]

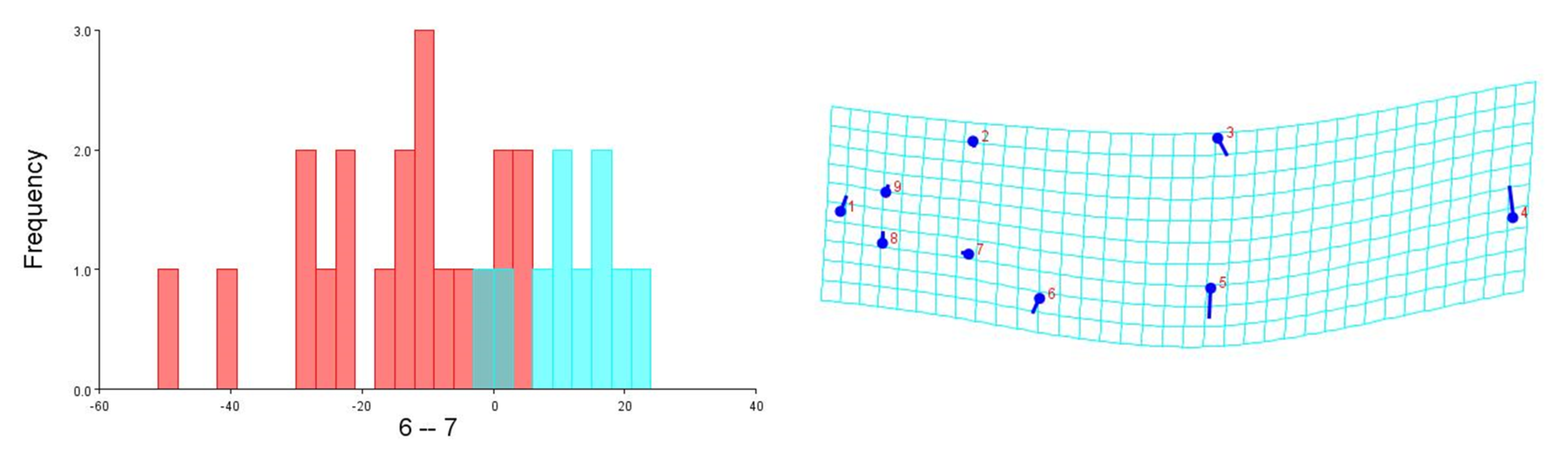

Supplement: Supplemental Information 5 [file peerj-10-13290-s005.png]

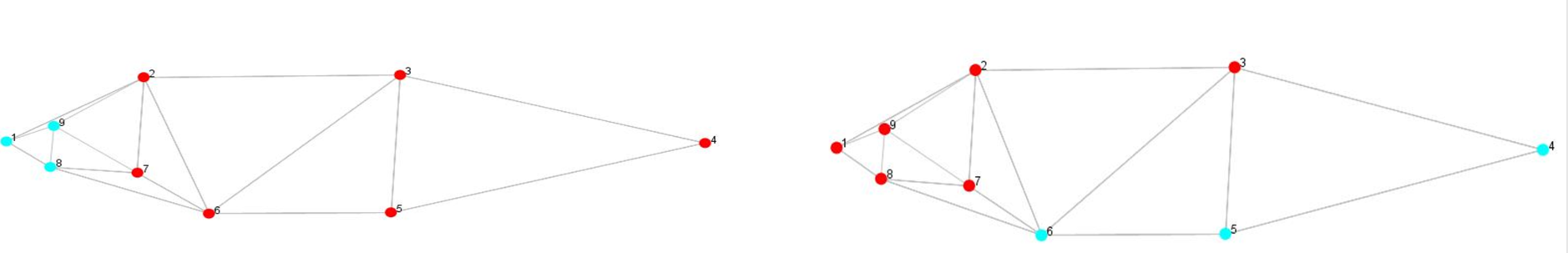

Supplement: Supplemental Information 6 [file peerj-10-13290-s006.png]

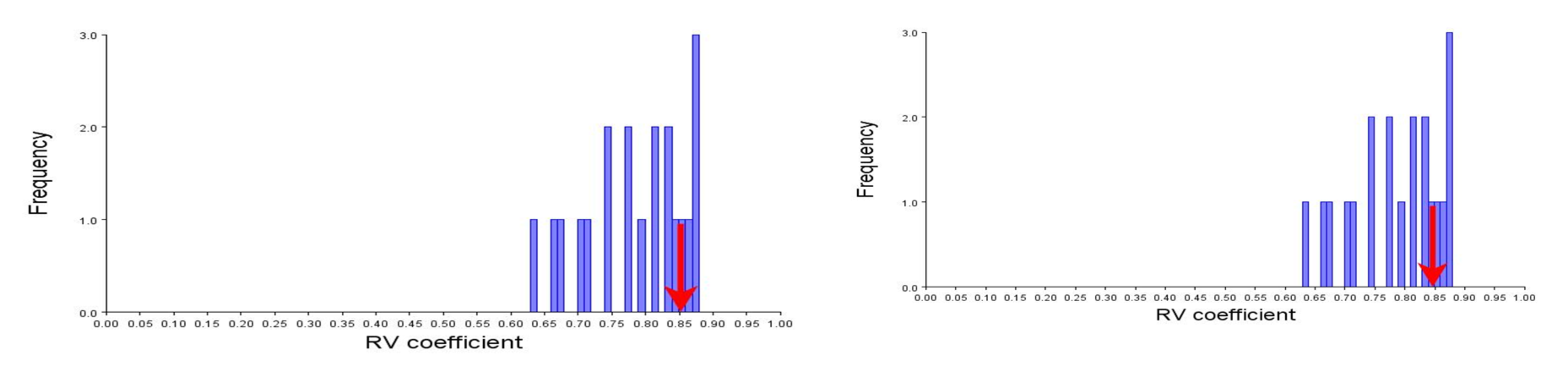

Supplement: Supplemental Information 7 [file peerj-10-13290-s007.png]

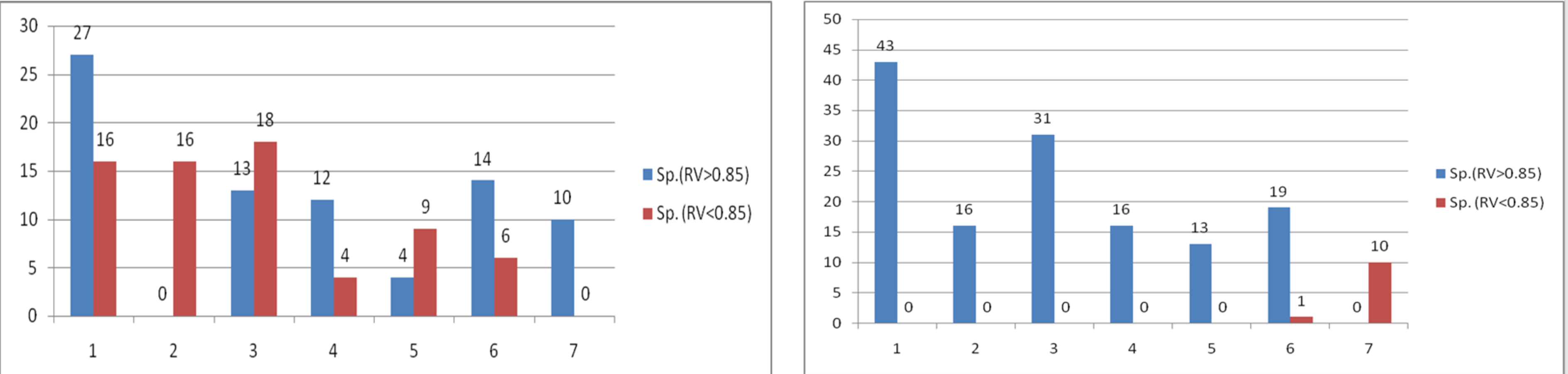

Supplement: Supplemental Information 8 — 1. Son 2. Tons, 3. Ken 4. Brahmaputra 5. Ganga 6. Gomti 7. Gandak [file peerj-10-13290-s008.png]
