# Supplementary material for: Phenotypic variation of Chitala chitala (Hamilton, 1822) from Indian rivers using truss network and geometric morphometrics
Source: PeerJ. 2022 Apr 18;10:e13290. doi: 10.7717/peerj.13290 (PMC9022642; doi:10.7717/peerj.13290)
Supplement: Supplemental Information 9 [file peerj-10-13290-s009.docx]

**Supplemental Table 1: Wilks' Lamda in testing of functions derived from discriminant analysis on principal components (DAPC)**

| **Test of Function(s)** | **Wilks' Lambda** | **Chi-square** | **Degrees of freedom** | **Significance (p<0.05)** |
| --- | --- | --- | --- | --- |
| 1 through 6 | 0.02 | 485.35 | 204 | 0.00 |
| 2 through 6 | 0.06 | 356.79 | 165 | 0.00 |
| 3 through 6 | 0.14 | 248.93 | 128 | 0.00 |
| 4 through 6 | 0.30 | 153.77 | 93 | 0.00 |
| 5 through 6 | 0.53 | 80.41 | 60 | 0.04 |
