# Supplementary material for: Phenotypic variation of Chitala chitala (Hamilton, 1822) from Indian rivers using truss network and geometric morphometrics
Source: PeerJ. 2022 Apr 18;10:e13290. doi: 10.7717/peerj.13290 (PMC9022642; doi:10.7717/peerj.13290)
Supplement: Supplemental Information 10 [file peerj-10-13290-s010.docx]

**Supplemental Table 2: Eigenvalues of functions derived from discriminant analysis on principal components (DAPC)**

| **Test of Function(s)** | **Eigenvalue** | **Percent of Variance** | **Cumulative Percentage** | **Canonical Correlation** |
| --- | --- | --- | --- | --- |
| 1 through 6 | 1.74 | 30.45 | 30.45 | 0.80 |
| 2 through 6 | 1.33 | 23.27 | 53.72 | 0.76 |
| 3 through 6 | 1.11 | 19.40 | 73.13 | 0.73 |
| 4 through 6 | 0.78 | 13.61 | 86.73 | 0.66 |
| 5 through 6 | 0.53 | 9.31 | 96.04 | 0.59 |
