# Supplementary material for: Phenotypic variation of Chitala chitala (Hamilton, 1822) from Indian rivers using truss network and geometric morphometrics
Source: PeerJ. 2022 Apr 18;10:e13290. doi: 10.7717/peerj.13290 (PMC9022642; doi:10.7717/peerj.13290)
Supplement: Supplemental Information 11 [file peerj-10-13290-s011.docx]

**Supplemental Table 3: Truss variables-shape variation on Coefficient of Variation (CV %) analysis across rivers**

| **SL. No** | **Location** | **Coefficient of Variation (CV %) of Truss variables over sampling locations** | | | |
| --- | --- | --- | --- | --- | --- |
|  |  | **1_5** | **2_3** | **5_7** | **5_9** |
|  | Son | 0.7 | 1.78 | 1.92 | 1.15 |
|  | Tons | 1.29 | 1.58 | 1.72 | 1.66 |
|  | Ken | 0.71 | 1.21 | 1.53 | 0.9 |
|  | Brahmaputra | 0.97 | 1.25 | 1.46 | 1.25 |
|  | Ganga | 0.93 | 1.04 | 1.23 | 1.13 |
|  | Gomti | 1.63 | 2.36 | 2.68 | 2.25 |
|  | Gandak | 1.1 | 1.45 | 1.56 | 1.25 |

1_5: Distance between anterior tip of snout at upper jaw and point perpendicular to dorsal fin origin; 2_3: Distance between posterior most aspect of neurocranium and dorsal fin origin; 5_7: Distance between point perpendicular to dorsal fin origin and pectoral fin insertion; 5_9: Distance between point perpendicular to dorsal fin origin and point perpendicular to posterior end of maxilla
