# Supplementary material for: Phenotypic variation of Chitala chitala (Hamilton, 1822) from Indian rivers using truss network and geometric morphometrics
Source: PeerJ. 2022 Apr 18;10:e13290. doi: 10.7717/peerj.13290 (PMC9022642; doi:10.7717/peerj.13290)
Supplement: Supplemental Information 12 [file peerj-10-13290-s012.docx]

**Supplemental Table 4: Warp density score (WDS) of shape over principal components (PC1, PC2) for shape-based variations**

| Sl.No | Location | WDS-PC1 | WDS-PC2 |
| --- | --- | --- | --- |
| 1 | Son | 18.00 | 3.63 |
| 2 | Tons | -2.00 | -25.25 |
| 3 | Ken | -5.32 | -3.03 |
| 4 | Brahmaputra | 21.40 | 20.20 |
| 5 | Ganga | -50.50 | -3.18 |
| 6 | Gomti | 9.08 | 6.56 |
| 7 | Gandak | 2.32 | -33.67 |
