# Supplementary material for: Phenotypic variation of Chitala chitala (Hamilton, 1822) from Indian rivers using truss network and geometric morphometrics
Source: PeerJ. 2022 Apr 18;10:e13290. doi: 10.7717/peerj.13290 (PMC9022642; doi:10.7717/peerj.13290)
Supplement: Supplemental Information 13 [file peerj-10-13290-s013.docx]

**Supplemental Table 5: Procrustes ANOVA for centroid size-based differences**

| **Effect** | **SS** | **MS** | **df** | **F** | **Significant p (0.05)** |
| --- | --- | --- | --- | --- | --- |
| Centroid size-Location | 7768.49 | 1294.74 | 6 | 10.40 | <0.0001 |
| Error | 17676.35 | 124.48 | 142 |  |  |
