# Supplementary material for: Phenotypic variation of Chitala chitala (Hamilton, 1822) from Indian rivers using truss network and geometric morphometrics
Source: PeerJ. 2022 Apr 18;10:e13290. doi: 10.7717/peerj.13290 (PMC9022642; doi:10.7717/peerj.13290)
Supplement: Supplemental Information 15 [file peerj-10-13290-s015.docx]

**Supplemental Table 7. Generalized Procrustes shape variation associated with principal components from principal component analysis**

| **Principal Component** | **Eigenvalues** | **% Variance** | **Cumulative %** |
| --- | --- | --- | --- |
| 1. | 0.00281289 | 67.530 | 67.530 |
| 2. | 0.00040837 | 9.804 | 77.334 |
| 3. | 0.00035859 | 8.609 | 85.942 |
| 4. | 0.00023630 | 5.673 | 91.615 |
| 5. | 0.00012954 | 3.110 | 94.725 |
| 6. | 0.00007888 | 1.894 | 96.619 |
| 7. | 0.00004218 | 1.013 | 97.632 |
