# Supplementary material for: Phenotypic variation of Chitala chitala (Hamilton, 1822) from Indian rivers using truss network and geometric morphometrics
Source: PeerJ. 2022 Apr 18;10:e13290. doi: 10.7717/peerj.13290 (PMC9022642; doi:10.7717/peerj.13290)
Supplement: Supplemental Information 16 [file peerj-10-13290-s016.docx]

**Supplemental Table 8. Procrustes distance (mean value) on principal components (PC-1&2) through PCA (relative warps) analysis**

| **Locations** | **Mean Procrustes distance** | |
| --- | --- | --- |
|  | **PC1** | **PC2** |
| Son | -0.01 | -0.01 |
| Tons | -0.03 | 0.01 |
| Ken | 0.02 | 0.01 |
| Brahmaputra | -0.03 | 0.00 |
| Ganga | -0.02 | 0.01 |
| Gomti | 0.00 | 0.00 |
| Gandak | 0.07 | 0.00 |
