# Supplementary material for: Phenotypic variation of Chitala chitala (Hamilton, 1822) from Indian rivers using truss network and geometric morphometrics
Source: PeerJ. 2022 Apr 18;10:e13290. doi: 10.7717/peerj.13290 (PMC9022642; doi:10.7717/peerj.13290)
Supplement: Supplemental Information 17 [file peerj-10-13290-s017.docx]

**Supplemental Table 9. The landmarks over body of fish specimen partitioned into two subsets with relative variance (RV) coefficient.**

| Sl. No. | Subset I | Subset II | RV coefficient |
| --- | --- | --- | --- |
| 1 | 2,3,4,5,6,7 | 1,8,9 | 0.85 |
| 2 | 1,2,3,7,8,9 | 4,5,6 | 0.85 |
